# The BALB/c.*mdx*62 mouse exhibits a dystrophic muscle pathology and is a model of Duchenne muscular dystrophy

**DOI:** 10.1242/dmm.050502

**Published:** 2024-05-03

**Authors:** Kristy Swiderski, Audrey S. Chan, Marco J. Herold, Andrew J. Kueh, Jin D. Chung, Justin P. Hardee, Jennifer Trieu, Annabel Chee, Timur Naim, Paul Gregorevic, Gordon S. Lynch

**Affiliations:** ^1^ Centre for Muscle Research, Department of Anatomy and Physiology, The University of Melbourne, Melbourne, VIC 3010, Australia; ^2^ The Walter and Eliza Hall Institute of Medical Research, Parkville, VIC 3052, Australia; ^3^ Department of Medical Biology, The University of Melbourne, Melbourne, VIC 3052, Australia; ^4^ Olivia Newton-John Cancer Research Institute, Heidelberg, VIC 3084, Australia; ^5^ School of Cancer Medicine, La Trobe University, Heidelberg, VIC 3084, Australia

**Keywords:** Muscular dystrophy, Skeletal muscle, Bone, Pathophysiology, Preclinical, Genetic modifier

## Abstract

Duchenne muscular dystrophy (DMD) is a devastating monogenic skeletal muscle-wasting disorder. Although many pharmacological and genetic interventions have been reported in preclinical studies, few have progressed to clinical trials with meaningful benefit. Identifying therapeutic potential can be limited by availability of suitable preclinical mouse models. More rigorous testing across models with varied background strains and mutations can identify treatments for clinical success. Here, we report the generation of a DMD mouse model with a CRISPR-induced deletion within exon 62 of the dystrophin gene (*Dmd*) and the first generated in BALB/c mice. Analysis of mice at 3, 6 and 12 months of age confirmed loss of expression of the dystrophin protein isoform Dp427 and resultant dystrophic pathology in limb muscles and the diaphragm, with evidence of centrally nucleated fibers, increased inflammatory markers and fibrosis, progressive decline in muscle function, and compromised trabecular bone development. The BALB/c.*mdx*62 mouse is a novel model of DMD with associated variations in the immune response and muscle phenotype, compared with those of existing models. It represents an important addition to the preclinical model toolbox for developing therapeutic strategies.

## Teaser

**Summary:** BALB/c.*mdx*62 mice are a novel model of Duchenne muscular dystrophy with a deletion in exon 62 of the *Dmd* gene, and exhibit dystrophic skeletal muscle pathology and an associated bone phenotype.

## INTRODUCTION

Duchenne muscular dystrophy (DMD) is an X-linked genetic muscle-wasting disorder arising from mutations in the dystrophin gene (*DMD*) resulting in aberrant expression of the dystrophin protein. It affects one in 3500-6000 live male births worldwide, with an overall prevalence of less than ten cases per 100,000 males (Bushby et al., 2010; Duan et al., 2021; Hoffman et al., 1987). In skeletal muscle, the dystrophin protein isoform Dp427 forms part of a sarcolemmal multimeric protein complex termed the dystrophin glycoprotein complex (DGC), which links the extracellular matrix to the internal muscle fiber actin cytoskeleton to mediate the forces of contraction (Ervasti and Campbell, 1993) and acts a hub for signaling activity that may regulate muscle proteostasis (Acharyya et al., 2005; Garbincius and Michele, 2015; Langenbach and Rando, 2002; Xiong et al., 2009). In the absence of dystrophin, the DGC fails to accumulate at the sarcolemma, resulting in membrane fragility, microtearing upon contraction and initiating events that ultimately lead to fiber degeneration and muscle wasting.

Despite the cause of DMD being attributed to mutations in dystrophin after identification of the *DMD* gene in 1987 (Hoffman et al., 1987), treatment options have been limited to corticosteroid therapy via administration of prednisone/prednisolone, deflazacort [approved by the US Food and Drug Administration (FDA) in 2017] and, more recently, vamorolone (AGAMREE; approved by the FDA and European Medicines Agency in 2023). However, although corticosteroid therapies delay disease progression and improve ambulation and lifespan in patients with DMD, their chronic use is associated with significant side effects including impacts on height, body mass and bone health, although these effects may be somewhat reduced with vamorolone (Grounds and Lloyd, 2023). The past decade has seen the advent of the first dystrophin restoration therapies, with FDA approval of exon-skipping drugs: Exondys 51 (eteplirsen) in 2016 for exon 51 skipping; Vyondys 53 (golodirsen) in 2019 and Viltepso (viltolarsen) in 2020 for exon 53 skipping; and Amondys 45 (casimersen) in 2021 for exon 45 skipping. Although a significant and exciting advance for DMD, these therapies remain expensive and restricted to patients with specific variants. The FDA also recently approved adeno-associated virus (AAV) gene therapy for DMD in 2023 (Elevidys), but the long-term clinical benefits of these gene replacement therapies have yet to be evaluated. There remains no cure or effective treatment for a large majority of patients (Hoffman, 2020).

As a monogenic disorder, the ultimate cure for DMD will come from correcting the underlying genetic defect, and preclinical studies in animal models of DMD are essential for testing various viral and non-viral genetic strategies to restore dystrophin expression. Such studies using compounds to induce read-through of premature stop codons or skipping of mutated exons (Barton-Davis et al., 1999; Lu et al., 2003) and viral vectors to re-express dystrophin mini-genes have rescued the dystrophic phenotype in murine and canine models (Blankinship et al., 2006; Bostick et al., 2012; Duan, 2018; Fabb et al., 2002; Gregorevic et al., 2004; Liu et al., 2005; Wang et al., 2007), with some proceeding to clinical trials and the approval of genetic modifiers to treat DMD (Bitetti et al., 2021; Dunant et al., 2003; Malik et al., 2010; Mercuri et al., 2020; Michorowska, 2021; Pasca et al., 2022; Ruggiero et al., 2018). Therefore, rigorous preclinical models that recapitulate the dystrophic pathology in patients remain critical to developing treatments for DMD.

The C57BL/10ScSn *mdx* mouse, harboring a naturally occurring point mutation in exon 23 of the *Dmd* gene, is the most commonly studied preclinical model of DMD. Although these mice are genetically similar to patients with DMD, the mice have a mild phenotype owing to compensatory upregulation of a homologous protein, utrophin (Deconinck et al., 1998; Rafael et al., 1998; Tinsley et al., 1998). To generate a murine model lacking both dystrophin and utrophin, the utrophin gene (*Utrn*) was deleted from the C57BL/10ScSn *mdx* mouse, and although these *mdx*/*utrn^−/^*^−^ or *dko* mice are no longer genetically similar to patients with DMD, their phenotype more closely resembles the disease progression in patients (Deconinck et al., 1998). The C57BL/10ScSn *mdx* mouse has also been bred onto the DBA/2J mouse strain, referred to as the D2.*mdx* mouse. These mice also exhibit a more severe muscle pathology than that of C57BL/10ScSn *mdx* mice, which has been attributed to a mutation in the *Ltbp4* gene in the DBA/2J mouse that enhances TGFβ signaling and fibrosis (Coley et al., 2016).

As all these models originate from the C57LB/10ScSn *mdx* mouse, they contain the same dystrophin mutation in exon 23, which results in the absence of the full-length Dp427 dystrophin protein isoform but does not affect expression of other isoforms. To assess the impact of mutations across the gene, affecting different dystrophin isoforms, chemical mutagenesis has been utilized to generate the *mdx^2cv^*, *mdx^3cv^*, *mdx^4cv^* and *mdx^5cv^* mouse models (Cox et al., 1993). In addition, gene targeting to delete exon 52 in C57BL/6J mice (*mdx*52) resulted in the absence of the Dp427, Dp260 and Dp140 dystrophin isoforms with the expected dystrophic pathology (Araki et al., 1997).

Each of these models has advantages and disadvantages for use in preclinical studies, and there is no one perfect preclinical murine model for studying DMD. However, given the sheer number of DMD-causative mutations and the heterogeneity of the human population, something not generally modeled in mouse studies, the use of multiple models in combination provides a powerful approach to assess treatment efficacy in mice with different mutation locations, disease severity and genetic backgrounds. To this end, we created a novel dystrophin-deficient mouse using CRISPR/Cas9 in BALB/c mice, termed C.*mdx*^emDel9418-9425^ (BALB/c.*mdx*62; hereafter referred to as C.*mdx*62), with an 8 bp deletion in the coding sequence of the *Dmd* gene mapping to exon 62. In this study, we characterized the skeletal muscle and bone phenotypes of this mouse that identify it as a useful murine model of DMD.

## RESULTS

### Dystrophin-mutant mice lack Dp427 protein expression

**Fig. 1.**
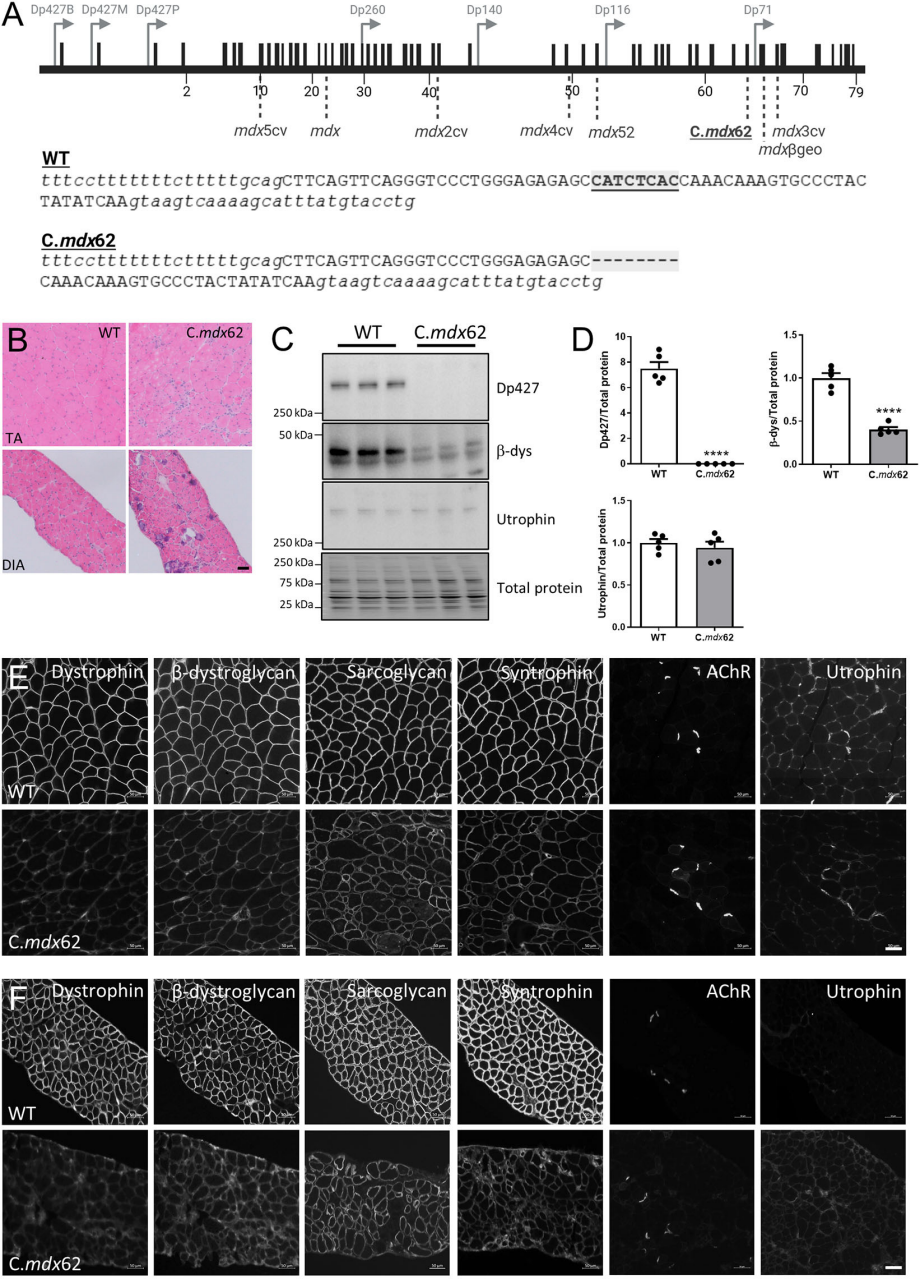
**C.*mdx*62 mice lack dystrophin and have reduced DGC protein expression at the sarcolemma of skeletal muscle fibers.** (A) Top: schematic of the dystrophin gene sequence. Black boxes represent exons and grey arrows show promoter locations for different dystrophin isoforms. Light grey dotted lines indicate locations of reported mutations in Duchenne muscular dystrophy (DMD) mouse models, including C.*mdx*62. Bottom: schematic of exon 62 (uppercase letters) flanked by intron 61 and intron 62 (lowercase letters) showing the 8 bp deletion within exon 62 in C.*mdx*62 mice (highlighted and underlined) relative to the WT sequence. Created with BioRender.com. (B) Representative Hematoxylin and Eosin-stained sections of tibialis anterior (TA) muscle and diaphragm (DIA) from 3-month-old WT and C.*mdx*62 mice (*n*=5 mice/genotype). Scale bar: 50 µm. (C) Representative western blots from lysates prepared from gastrocnemius muscles from 3-month-old WT and C.*mdx*62 mice (*n*=5 mice/genotype) were analyzed for dystrophin (Dp427), β-dystroglycan (β-dys) and utrophin protein expression, shown with total protein stain imaged on stain-free gels. (D) Levels of dystrophin (Dp427), β-dystroglycan and utrophin protein were quantified relative to total protein levels. Data are presented as mean±s.e.m. Statistical analysis was performed using a two-tailed unpaired Student's *t*-test. ****P<0.0001. (E,F) Representative sections of TA muscle (E) and diaphragm (F) from 3-month-old WT and C.*mdx*62 mice immunostained for dystrophin glycoprotein complex (DGC) proteins: dystrophin, β-dystroglycan, sarcoglycan, syntrophin, acetylcholine receptor (AChR) and utrophin (*n*=5 mice/genotype). Scale bars: 50 µm.

CRISPR/Cas9 technology was used with single guide RNAs (sgRNAs) designed to introduce point mutations in exon 62 of the *Dmd* gene, which encodes part of the WW domain of the dystrophin protein, a region of interest that directly regulates the interaction between dystrophin and β-dystroglycan (encoded by *Dag1*) (Swiderski et al., 2021, 2014). In screening for these mutations, a small insertion/deletion (indel) was identified in which 8 bp were deleted in exon 62, just upstream of the mutations reported in the *mdx^3cv^* and *mdx*^βgeo^ mouse models (Fig. 1A). Analysis of gene variants from patients at this site demonstrated an association with a DMD phenotype [Unique variants in the *DMD* gene, Global Variome shared Leiden Open Variation Database (LOVD), https://databases.lovd.nl/shared/variants/DMD/unique], and analysis with a reading frame checker indicated a frameshift mutation likely to result in degradation of the protein product. Histological analysis via Hematoxylin and Eosin staining confirmed the expected dystrophic pathology in the tibialis anterior (TA) muscles and diaphragms of 3-month-old mice (Fig. 1B), with the presence of inflammatory infiltrate, variable fiber sizes and centrally nucleated fibers. Western blotting confirmed the absence of the dystrophin Dp427 protein isoform from the gastrocnemius muscle, with a subsequent reduction in the overall expression of the β-dystroglycan protein (Fig. 1C,D). No change in the level of utrophin protein expression was detected (Fig. 1C,D). Western blotting extracts from the sciatic nerve confirmed the loss of Dp116 expression but maintenance of Dp71 and Dp40 expression as expected, based on the location of the mutation (Fig. S1). Immunofluorescence analyses confirmed the absence of dystrophin protein at the sarcolemma of fibers in the TA muscle (Fig. 1E) and diaphragm (Fig. 1F), with decreased sarcolemmal localization of the associated DGC proteins, β-dystroglycan, α-sarcoglycan (encoded by *Sgca*) and α1-syntrophin (encoded by *Snta1*) (Fig. 1E,F). Consistent with western blotting data, no increase in utrophin protein expression was observed in the TA muscles (Fig. 1E) or diaphragm (Fig. 1F) of C.*mdx*62 mice, with the protein signals localizing to the neuromuscular junction, as indicated by staining for the acetylcholine receptor (AChR).

### Dystrophin-mutant mice exhibit skeletal muscle hypertrophy

**Fig. 2.**
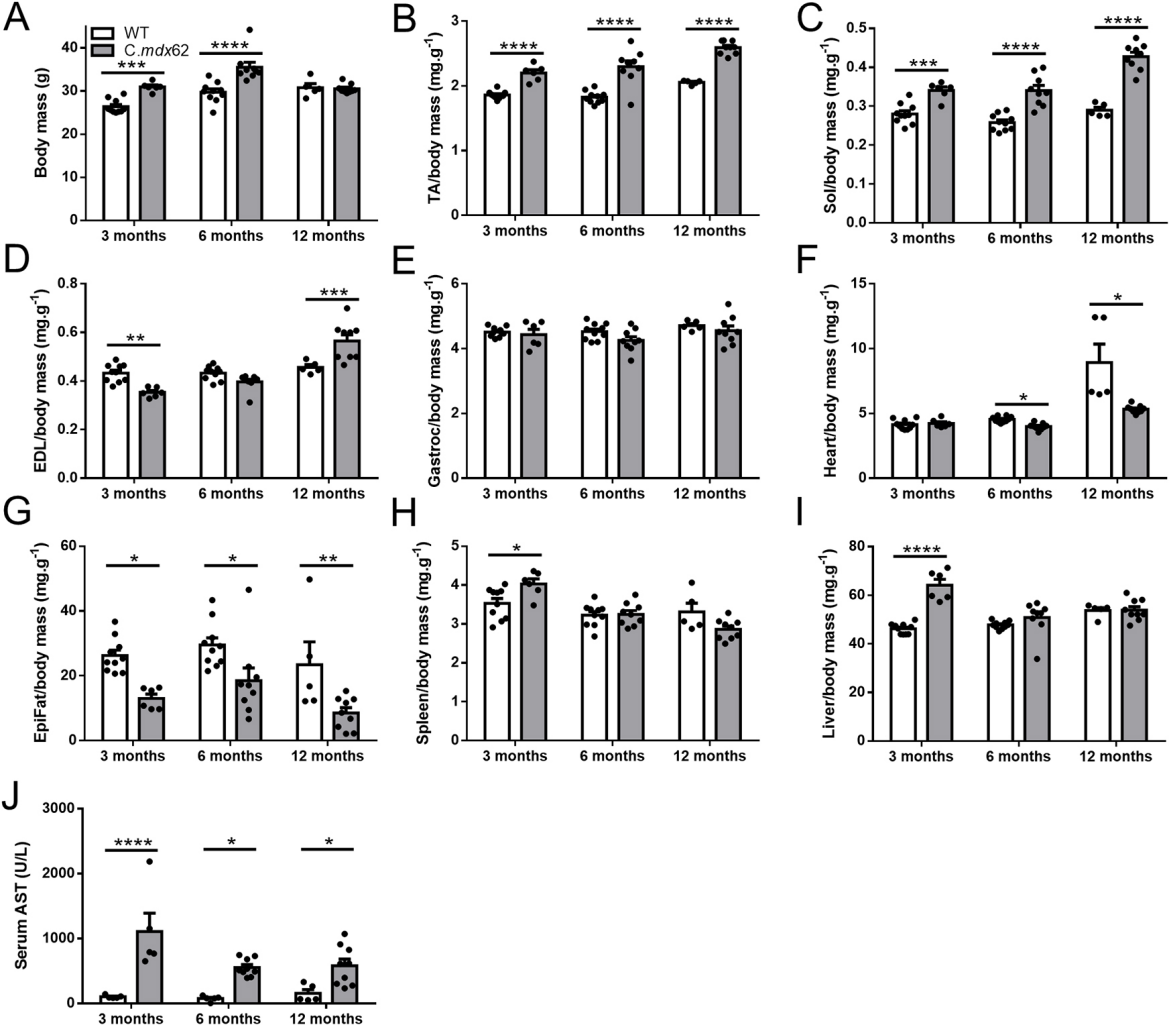
**C.*mdx*62 mice exhibit skeletal muscle hypertrophy, reduced epididymal fat mass and increased serum markers of muscle damage.** (A-J) Male WT and C.*mdx*62 mutant mice were killed at 3 months (*n*=10 and 6 mice/genotype, respectively), 6 months (*n*=10 and 9 mice/genotype, respectively) and 12 months (*n*=5 and 9 mice/genotype, respectively) of age, and assessed for body mass (A), as well as mass of the TA (B), soleus (C), extensor digitorum longus (EDL) (D) and gastrocnemius (E) muscles, heart (F), epididymal fat pad (G), spleen (H) and liver (I), relative to body mass. (J) Serum was assessed for aspartate aminotransferase (AST) levels. Data are presented as mean±s.e.m. Statistical analysis was performed using a two-way ANOVA with a post hoc Bonferroni's test, with the exception of the heart data, which were analyzed using a Mann–Whitney U test due to a non-normal distribution. *P<0.05, **P<0.01, ***P<0.001 and ****P<0.0001, relative to age-matched WT mice.

BALB/c wild-type (WT) and C.*mdx*62 mutant mice were killed at 3, 6 and 12 months of age to characterize the skeletal muscle phenotype. Relative to WT BALB/c mice, C.*mdx*62 mice tended to be heavier, with significantly greater body mass at 3 and 6 months of age, and no change in body mass at 12 months of age (Fig. 2A). Normalized masses of TA and soleus muscles from C.*mdx*62 mice were greater than those from WT mice at all ages studied (Fig. 2B,C). Extensor digitorum longus (EDL) muscles from C.*mdx*62 mice were smaller at 3 months of age, not different at 6 months of age, but heavier at 12 months of age relative to those of WT mice (Fig. 2D). Gastrocnemius muscles from C.*mdx*62 mice were not different in mass from those of WT mice at any age (Fig. 2E). Normalized heart mass was not different between WT and C.*mdx*62 mice at 3 months of age but was lower at 6 and 12 months of age for C.*mdx*62 mice relative to that for WT mice (Fig. 2F), even when the two abnormally large WT hearts were removed (Fig. S2). Consistent with previous reports in *mdx* mice, C.*mdx*62 mice had reduced epididymal fat mass at all ages studied (Fig. 2G). C.*mdx*62 mice had larger spleens (Fig. 2H) and livers (Fig. 2I) at 3 months of age, with no difference at 6 or 12 months of age. Serum analysis confirmed increased aspartate aminotransferase (AST) levels (Fig. 2J) in 3-, 6- and 12-month-old C.*mdx*62 mice relative to those in WT mice. Taken together, these data confirmed that C.*mdx*62 mice had typical features of dystrophic pathology.

### Muscles of C.*mdx*62 mice have an increased susceptibility to contraction-mediated damage

**Fig. 3.**
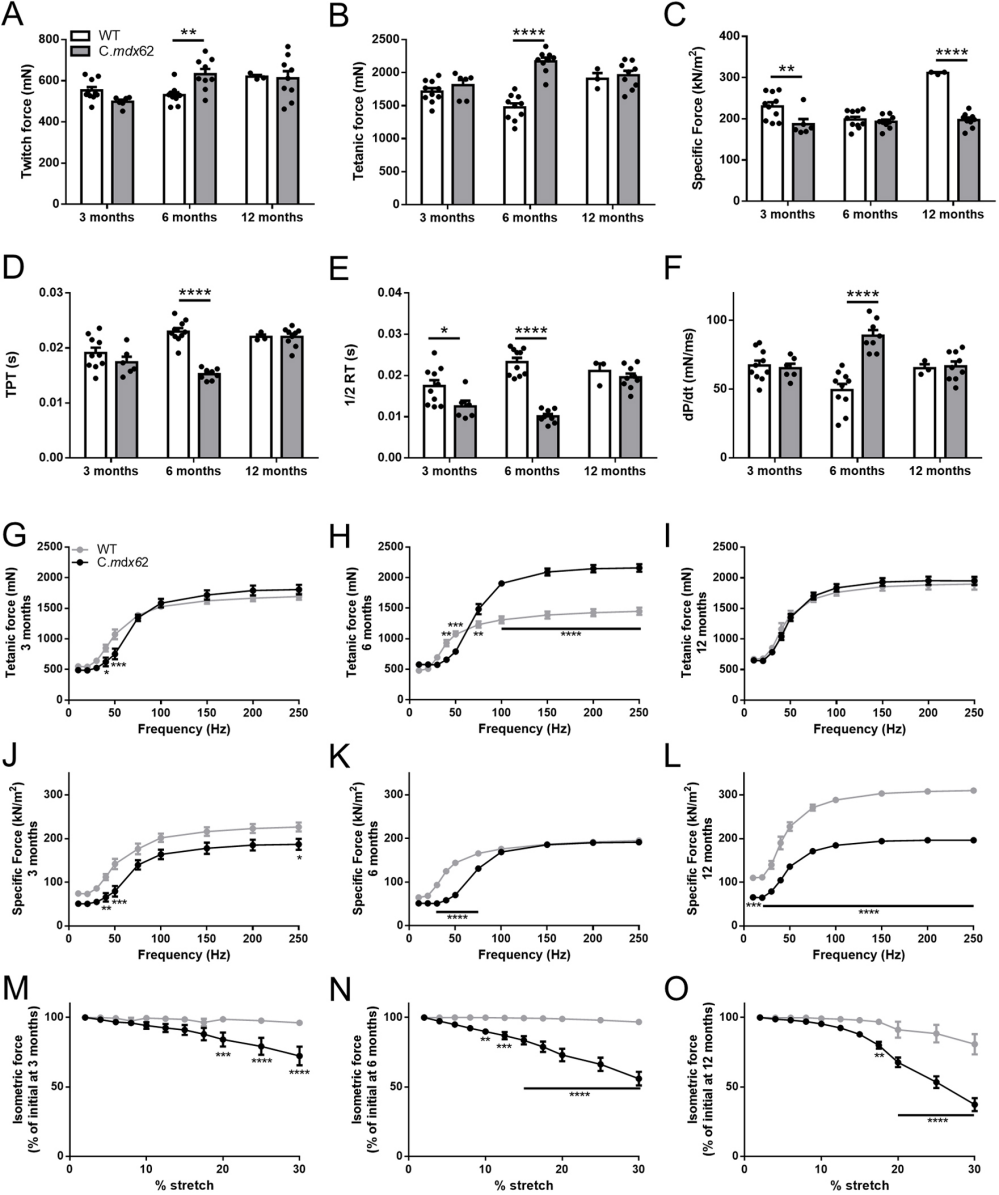
**Contractile properties of TA muscles from 3-, 6- and 12-month-old WT and C.*mdx*62 mice.** (A-F) Functional parameters of the TA muscle from male 3-, 6- and 12-month-old WT (*n*=10, 10 and 3 mice/age group, respectively) and C.*mdx*62 mutant (*n*=5, 9 and 9 mice/age group, respectively) mice were assessed *in situ* for maximum twitch force (A), maximum tetanic force (B), specific force (C), time to peak twitch (TPT) (D), one-half relaxation time (1/2 RT) (E) and maximum rate of twitch contraction (dP/dt) (F). Statistical analysis was performed using a two-way ANOVA with a post-hoc Bonferroni's test. (G-L) The frequency-force relationship to determine maximal force (G-I) and specific (normalized) force (J-L) were determined over a range of stimulation frequencies (10-250 Hz) at the various ages. (M-O) The cumulative force deficit associated with repeated lengthening contractions was used to assess the muscle susceptibility to contraction-mediated injury in 3-month-old (M), 6-month-old (N) and 12-month-old (O) mice. Data are presented as mean±s.e.m. Statistical analysis was performed using a repeated measures two-way ANOVA with a post hoc Bonferroni's test. *P<0.05, **P<0.01, ***P<0.001, ****P<0.0001, relative to age-matched WT mice.

Force production and the loss of force after repeated lengthening contractions were assessed in TA muscles from 3-, 6- and 12-month-old C.*mdx*62 mice relative to those from age-matched BALB/c WT mice. Twitch (Fig. 3A) and tetanic (Fig. 3B) forces were higher in C.*mdx*62 mice at 6 months but not at 3 or 12 months of age relative to those in WT mice. When normalized for changes in muscle cross-sectional area, specific force was lower in C.*mdx*62 mice relative to that in WT mice at 3 and 12 months but not at 6 months of age (Fig. 3C). Time to peak twitch (TPT; Fig. 3D) was faster only at 6 months of age, and one-half relaxation time (Fig. 3E) was faster at 3 and 6 months but not different at 12 months of age in C.*mdx*62 mice relative to that in WT mice, with the rate of twitch contraction (dP/dt) increased relative to WT only at 6 months of age (Fig. 3F). Analysis of fiber type proportions showed a shift towards a faster phenotype with a reduction in the proportion of type IIa fibers [detected by myosin heavy chain IIA (MYH2) immunostaining] in TA muscles of C.*mdx*62 mice at 6 and 12 months of age (Fig. S3A-D), increased size of type IIa (detected by MYH2 immunostaining) and IIb [detected by myosin heavy chain IIB (MYH4) immunostaining] fibers at 6 months and 12 months of age, respectively (Fig. S3A,E-G), but no change in fiber oxidative capacity based on succinate dehydrogenase (SDH) reaction intensity (Fig. S3H-K).

Frequency-force analyses revealed muscles from 3- and 6-month-old C.*mdx*62 mice produced less force at lower frequencies (Fig. 3G,H), but muscles from 6-month-old C.*mdx*62 mice produced greater force at higher frequencies (above 100 Hz), relative to muscles from WT mice (Fig. 3H). Force production was similar at all stimulation frequencies between 12-month-old WT and C.*mdx*62 mice (Fig. 3I). Normalized force production was reduced in muscles from C.*mdx*62 mice relative to that in muscles from WT mice at 3, 6 and 12 months of age (Fig. 3J-L; *P<*0.01, *P*<0.001, *P*<0.0001, genotype main effect, respectively), with post hoc analyses confirming reduced force production at lower frequencies at all ages (Fig. 3J-L) but only at higher frequencies at 3 months (Fig. 3J) and 12 months (Fig. 3L).

A key feature of dystrophin deficiency is a higher susceptibility to contraction-induced injury based on significantly higher force deficits after repeated lengthening contractions. When TA muscles were stretched by up to 30% of fiber length, the force deficit for 3-, 6- and 12-month-old WT mice averaged 4%, 3% and 19%, respectively, compared with TA muscles from 3-, 6- and 12-month-old C.*mdx*62 mice, with average force deficits of 24%, 44% and 61% from the initial (uninjured) force, respectively (Fig. 3M-O). It is worth noting that the function data for the 12-month-old WT mice were restricted to only three mice due to the unexpected death of two mice with calcified hearts. These data should therefore be considered as preliminary. Further comparisons between 12-month-old WT and C.*mdx*62 mice are warranted with additional WT mice. Overall, muscles from C.*mdx*62 mice were more susceptible to contraction-induced injury, and the force deficits were exacerbated with age.

### Skeletal muscles from C.*mdx*62 mice exhibit a dystrophic pathology

**Fig. 4.**
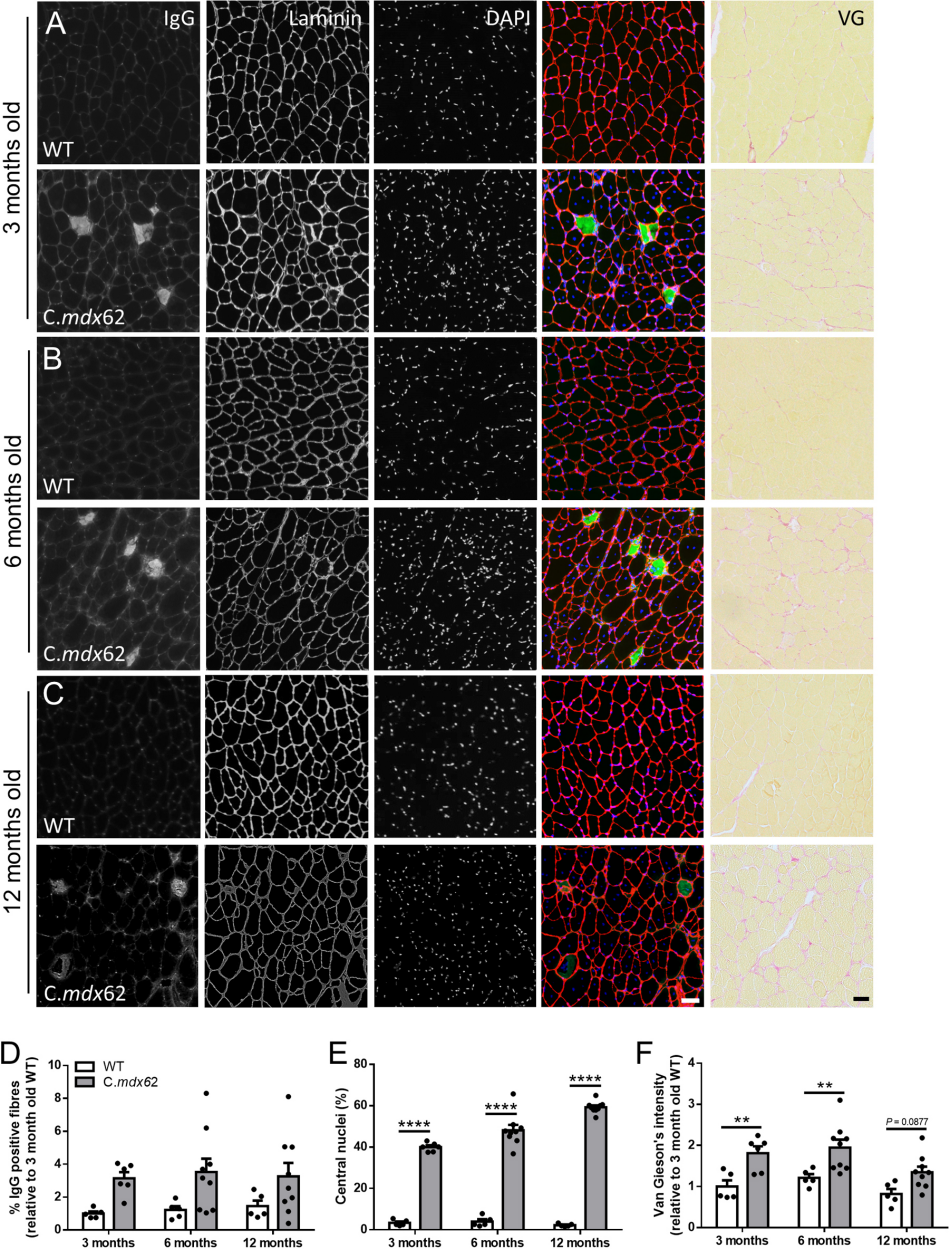
**IgG infiltration, central nuclei and fibrosis are increased in TA muscles of C.*mdx*62 mice.** Male WT and C.*mdx*62 mutant mice were killed at 3 months (*n*=5 and 6 mice/genotype, respectively) (A), 6 months (*n*=5 and 9 mice/genotype, respectively) (B) and 12 months (*n*=5 and 9 mice/genotype, respectively) (C) of age. (A-C) TA muscles were excised, sectioned and stained for IgG (green), laminin (red) and DAPI (blue), or stained with Van Gieson's stain. Scale bars: 50 µm. Sections were quantified to determine the percentage of IgG-infiltrated muscle fibers (D), the number of muscle fibers containing central nuclei (E) or the area of Van Gieson's staining (F). Data are presented as mean±s.e.m. Statistical analysis was performed using a two-way ANOVA with a post hoc Bonferroni's test. **P<0.01, ****P<0.0001, relative to age-matched WT mice.

The loss of dystrophin from skeletal muscle is well known to reduce membrane stability and result in ongoing cycles of degeneration and regeneration, evident from the increased prevalence of centrally nucleated fibers. To assess these parameters in C.*mdx*62 mice, immunoglobulin G (IgG) infiltration, central nucleation and fibrosis were assessed in the TA muscle and diaphragm. The percentage of IgG-positive fibers was increased in TA muscles of C.*mdx*62 mice (*P<*0.01, genotype main effect), with a trend towards a higher proportion at 3 months (*P=*0.16; Fig. 4A,D), 6 months (*P=*0.07; Fig. 4B,D) and 12 months (*P=*0.22; Fig. 4C,D), relative to that in WT mice. Immunostaining with laminin and nuclear staining with DAPI detected very few centrally located fibers in TA muscles from WT mice at 3 months (Fig. 4A,E), 6 months (Fig. 4B,E) and 12 months of age (Fig. 4C,E). Conversely, the proportion of fibers with centrally located nuclei increased to 40% in TA muscles of C.*mdx*62 mice at 3 months (Fig. 4A,E), 48% at 6 months (Fig. 4B,E) and 59% at 12 months (Fig. 4C,E), within the expected range reported in *mdx* mice (Coley et al., 2016) and as outlined in the standard operating protocols published by TREAT-NMD (SOP DMD_M.1.2.001, https://www.treat-nmd.org/wp-content/uploads/2023/07/MDX-DMD_M.1.2.001.pdf). Similarly, fibrosis was higher at 3 months (Fig. 4A,F) and 6 months (Fig. 4B,F) in TA muscles of C.*mdx*62 mice relative to that in WT mice, with a similar trend towards an increase at 12 months (*P=*0.0877; Fig. 4C,F). Analysis of laminin-stained sections also confirmed an increase in myofiber size at 6 and 12 months of age in TA muscles of C.*mdx*62 mice (Fig. S4A), which was attributed to the increased proportion of both smaller and larger muscle fibers at all ages (Fig. S4B-D).

**Fig. 5.**
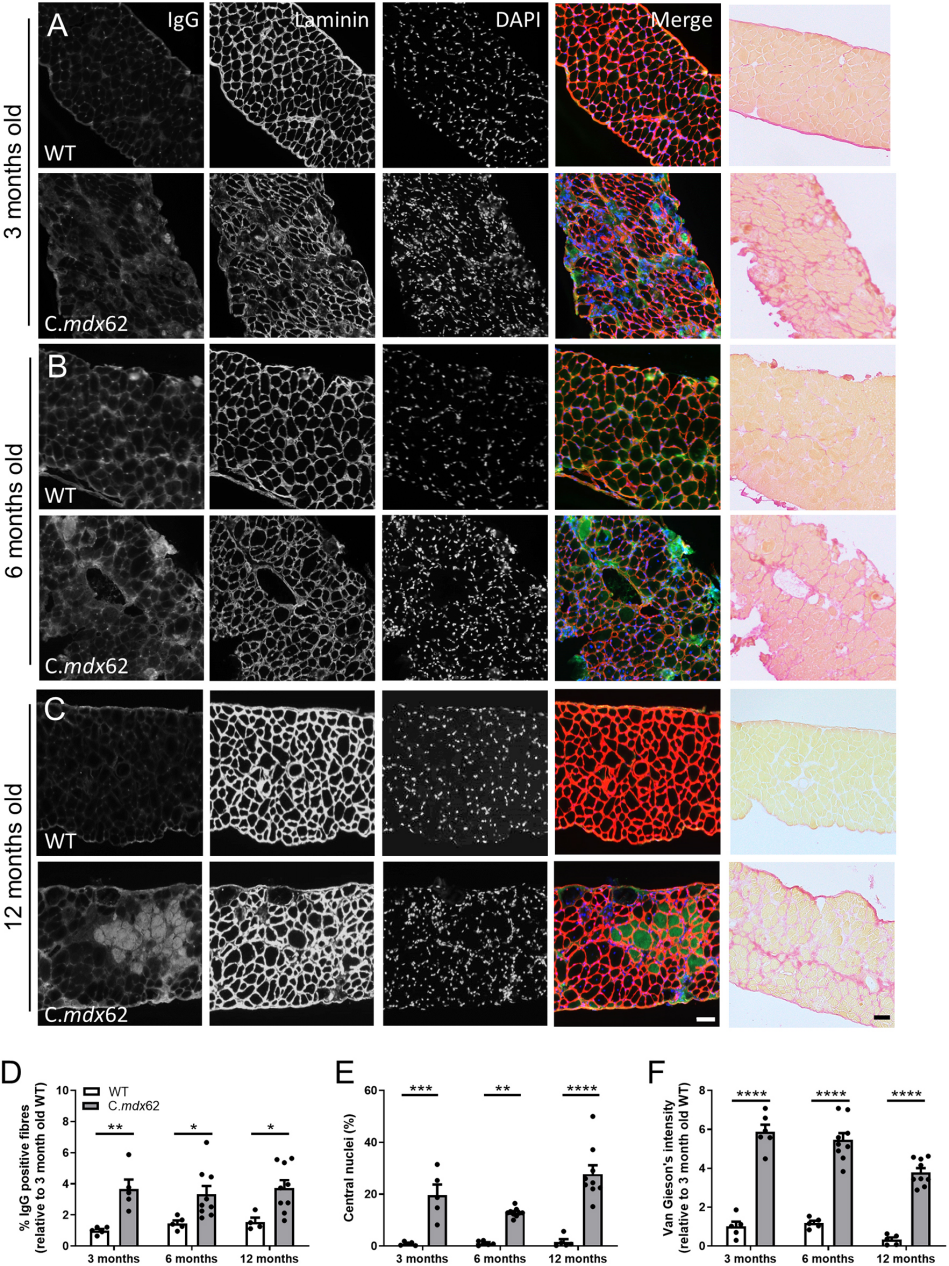
**IgG infiltration, central nuclei and fibrosis are increased in the diaphragm of C.*mdx*62 mice.** Male WT and C.*mdx*62 mutant mice were killed at 3 months (*n*=5 and 6 mice/genotype, respectively) (A), 6 months (*n*=5 and 9 mice/genotype, respectively) (B) and 12 months (*n*=5 and 9 mice/genotype, respectively) (C) of age. (A-C) Diaphragm muscles were excised, sectioned, and stained for IgG (green), laminin (red) and DAPI (blue), or stained with Van Gieson's stain. Scale bars: 50 µm. (D-F) Sections were quantified to determine the percentage of IgG-infiltrated muscle fibers (D), the number of muscle fibers containing central nuclei (E) or the area of Van Gieson's staining (F). Data are presented as mean±s.e.m. Statistical analysis was performed using a two-way ANOVA with a post hoc Bonferroni's test. *P<0.05, **P<0.01, ***P<0.001, ****P<0.0001, relative to age-matched WT mice.

In the diaphragm, the proportions of IgG-positive fibers (Fig. 5A-D), central nuclei (Fig. 5A-C,E) and fibrosis (Fig. 5A-C,F), were significantly higher in C.*mdx*62 mice relative to those in WT mice at all ages. Taken together, these data confirm significant dystrophic pathology up to 12 months of age in the limb and diaphragm muscles of C.*mdx*62 mice.

As dystrophic pathology also affects the heart, gene and protein expression markers of cardiac damage were assessed in WT and C.*mdx*62 mice at 3, 6 and 12 months of age. Western blotting analysis of phospholamban (encoded by *Pln*) phosphorylation showed a trend for increased phosphorylation in hearts from 3-month-old C.*mdx*62 mice (*P=*0.07; Fig. S5A), and reduced phosphorylation in hearts from 6-month-old C.*mdx*62 mice, relative to that in WT mice (Fig. S5A). Phospholamban phosphorylation was reduced in WT and C.*mdx*62 hearts at 12 months of age (Fig. S5A). Taken together, these data support earlier reductions in sarcoendoplasmic reticulum calcium ATPase (SERCA) function in the hearts of C.*mdx*62 mice relative to that in WT mice (Voit et al., 2017). Consistent with previous reports in BALB/c mice, two of the 12-month-old mice had calcified atria, which was not observed in any of the C.*mdx*62 mice (observed by A.C. during dissection of animals, data not shown). Gene expression analyses of markers of osteogenesis showed a main effect for increased *Runx2* expression with both increasing age and in C.*mdx*62 mice (*P<*0.05, age main effect; *P<*0.01, genotype main effect; Fig. S4B), but no change in either *Ocn* (officially known as *Bglap*) (Fig. S5C) or *Alpl* (Fig. S5D) expression. Markers of inflammation and fibrosis showed no changes in expression of *F4/80* (*Adgre1*) (Fig. S5E), *TGFβ* (*Tgfb1*) (Fig. S5F), *Col1a1* (Fig. S5G), *Col3a1* (Fig. S5H) or *Col6a1* (Fig. S5I). These preliminary findings suggest that the C.*mdx*62 mouse has a cardiac phenotype from 6 months of age, but more extensive histological and functional analyses are required to assess this pathology.

### Myofiber ossification in the diaphragm of C.*mdx*62 mice

**Fig. 6.**
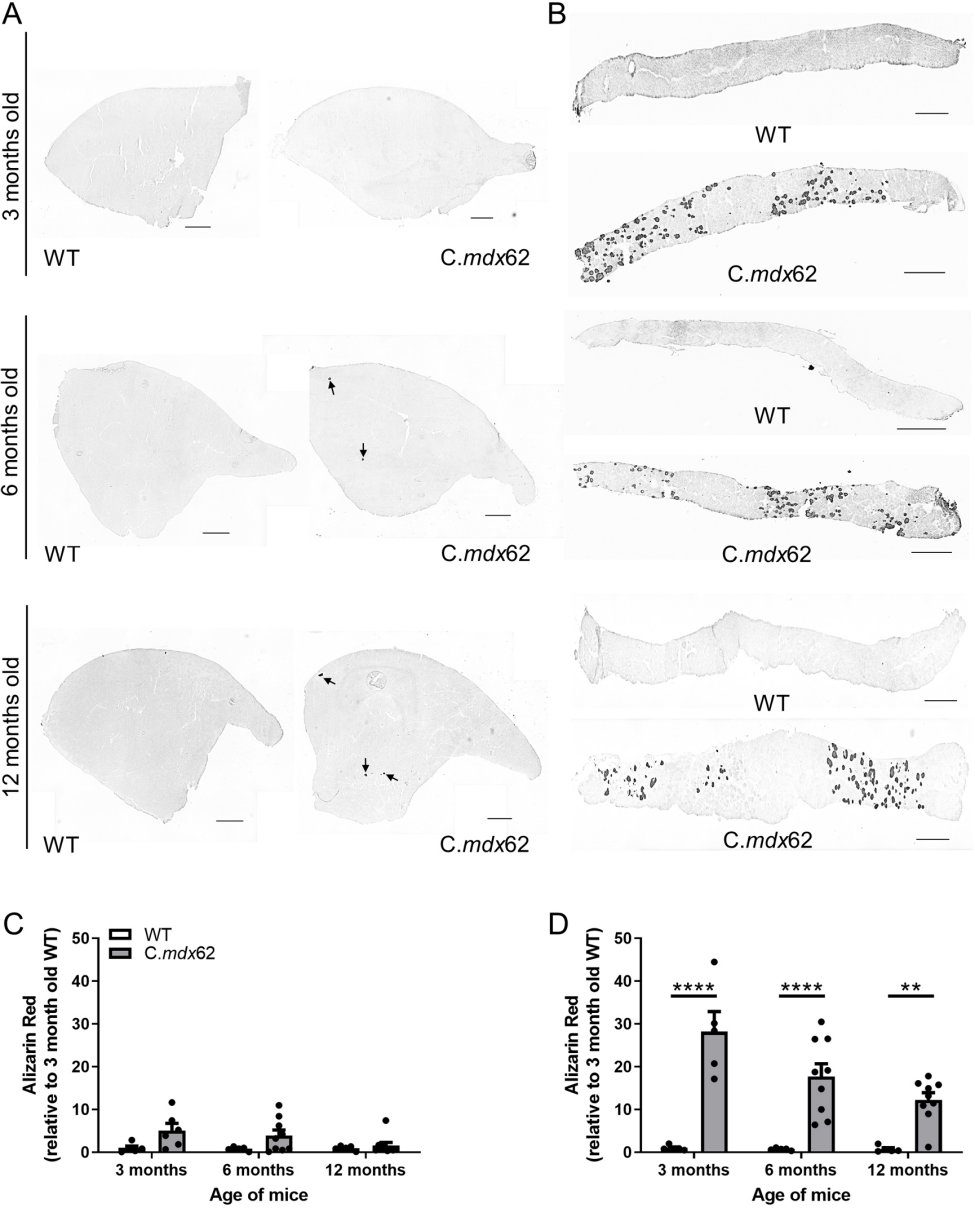
**Alizarin Red staining shows heterotopic ossification in the TA and diaphragm muscles of C.*mdx*62 mice.** Male WT and C.*mdx*62 mutant mice were killed at 3 months (*n*=5 and 6 mice/genotype, respectively), 6 months (*n*=5 and 9 mice/genotype, respectively) and 12 months (*n*=5 and 9 mice/genotype, respectively) of age. (A,B) TA (A) and diaphragm (B) muscles were excised, sectioned and stained with Alizarin Red for the presence of calcification. Arrows in A indicate areas of calcification in TA muscles from C.*mdx*62 mice. Scale bars: 500 µm. (C,D) Sections were quantified to determine the intensity of Alizarin Red staining in TA (C) and diaphragm (D) sections. Data are presented as mean±s.e.m. Statistical analysis was performed using a two-way ANOVA with a post hoc Bonferroni's test. **P<0.01, ****P<0.0001, relative to age-matched WT mice.

Calcifications within muscle have been reported in the more severely affected diaphragm, and in mouse models of DMD missing multiple dystrophin isoforms (Young et al., 2020). To determine whether calcification was evident, TA (Fig. 6A) and diaphragm (Fig. 6B) muscles from 3-, 6- and 12-month-old WT and C.*mdx*62 mice were stained with Alizarin Red. Calcification was not observed in TA or diaphragm muscles from WT mice, but some areas of calcification were evident in TA muscles from C.*mdx*62 mice at all ages (*P<*0.01, genotype main effect; Fig. 6A,C). Significant calcification was present in the diaphragm of C.*mdx*62 mice at 3, 6 and 12 months of age, which was reduced with increasing age (*P<*0.05, age main effect; Fig. 6B,D).

### C.*mdx*62 mice exhibit deficits in trabecular bone development

**Fig. 7.**
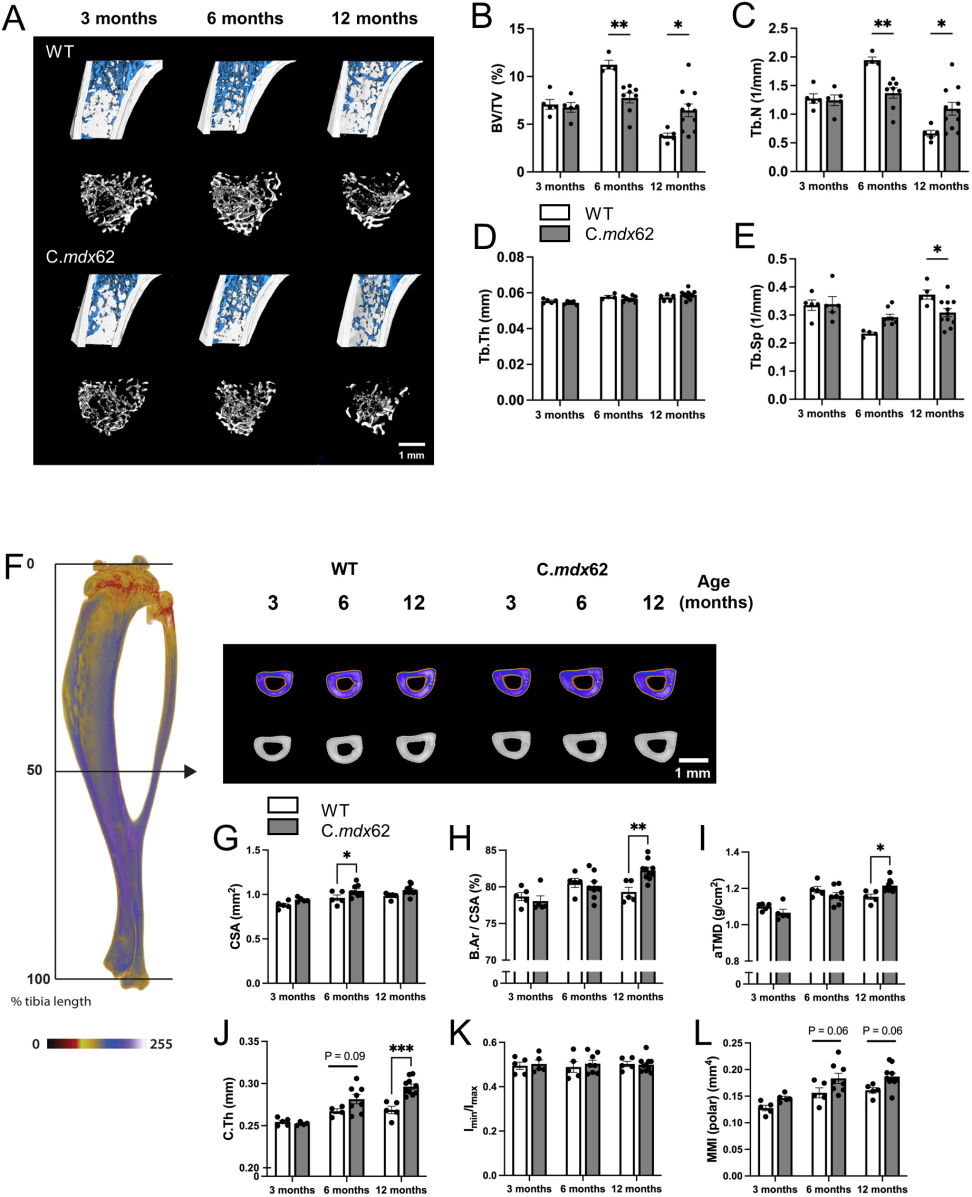
**Dystrophin deficiency delays trabecular bone mass accrual and alters cortical bone structure in C.*mdx*62 mice.** Tibial bones were taken from male WT and C.*mdx*62 mutant mice killed at 3 (*n*=5 and 5 mice/group, respectively), 6 (*n*=5 and 8 mice/group, respectively) and 12 (*n*=5 and 10 mice/group, respectively) months of age. (A) Representative images for WT and C.*mdx*62 mice across 3, 6 and 12 months of age, with trabecular bone shown in blue (top row) or white (bottom row) for each genotype. Scale bar: 1 mm. (B-E) Trabecular bone volume ratio (BV/TV, as a percentage) (B), trabecular number (Tb.N, 1/mm) (C), trabecular thickness (Tb.Th, in mm) (D) and trabecular separation (Tb.Sp, 1/mm) (E). Data are represented as mean±s.e.m. (F) Representative reconstructions are shown for WT and C.*mdx*62 mice at the tibial mid-diaphysis (50% of bone length), with a pseudodensity filter applied (top row). Scale bar: 1 mm. (G-L) Cross-sectional area (CSA, in mm^2^) (G), normalized bone area (B.Ar as a percentage of CSA) (H), areal tissue mineral density (aTMD, in g/cm^2^) (I), cortical thickness (C.Th, in mm) (J), bone shape ratio (ratio between minimum and maximum principal moments of inertia, I_min_/I_max_) (I) and polar moment of inertia (MMI, in mm^4^ (L). Data are represented as mean±s.e.m. Statistical significance was determined using two-way ANOVA followed by Bonferroni’s multiple comparisons test. *P<0.05, **P<0.01, ***P<0.005, relative to age-matched WT mice.

To characterize the bone phenotype in this model, we used micro-computed tomography to assess the trabecular bone within the proximal tibia (Fig. 7A). There was no difference in trabecular bone volume ratio (Fig. 7B) or trabecular number (Fig. 7C) between WT and C.*mdx*62 mice at 3 months of age. However, although trabecular bone development continued in WT mice between 3 and 6 months of age, trabecular bone mass and number were unchanged in C.*mdx*62 (Fig. 7B,C). At 12 months of age, as expected, WT mice exhibited age-related trabecular bone loss, evident from a decrease in trabecular bone volume ratio and number and increased trabecular separation (Fig. 7B-E). The loss of trabecular bone between 6 and 12 months of age was attenuated in C.*mdx*62 mice, and they exhibited more trabecular bone mass than that of age-matched WT mice.

We then assessed the cortical bone at the tibial mid-diaphysis (Fig. 7F). As for the trabecular bone, there was no difference in cortical bone parameters at 3 months of age. However, at 6 months of age, C.*mdx*62 mice had a significantly larger diaphysis (Fig. 7G) and, at 12 months of age, they had a greater normalized bone area (Fig. 7H), areal tissue mineral density (Fig. 7I) and cortical thickness (Fig. 7J) than those of the age-matched counterparts. There was no difference in shape ratio (Chadwick et al., 2018) (Fig. 7K) or resistance to torsional forces (Fig. 7L) at any age. Finally, and in light of our recent studies examining the influence of muscle mass on tibial geometry (Chan et al., 2023, 2021), we also found augmentation of the tibial crest in C.*mdx*62 mice due to the larger, adjacent TA muscle (Fig. S6). Collectively, these data indicate that skeletal development and structure are also affected in this murine model of DMD, with prominent effects on trabecular bone microstructure.

## DISCUSSION

Small animal models are essential tools for the development and preclinical testing of potential therapies for DMD. The gold standard and most widely used murine model is the *mdx* mouse, which genocopies the human disease but has only a mild skeletal muscle pathology, with the exception of the diaphragm (Swiderski and Lynch, 2021). A number of other murine models have been generated that either lack additional related proteins (e.g. utrophin), have mutations at different sites in the dystrophin gene (e.g. *mdx^2-5cv^*, *mdx52*, *mdx^βgeo^*) or result from crossing the *mdx* mouse onto different genetic backgrounds (e.g. D2.*mdx*) (Swiderski and Lynch, 2021). Importantly, no one model perfectly replicates the DMD pathology, but using a combination of models with different disease severities is a powerful approach for interrogating novel treatments. The generation of novel mouse models with varying mutation sites and a different genetic background, enhances these interrogative tools for DMD. To this end, we identified an indel during a CRISPR-based mutation screen resulting in an 8 bp deletion within exon 62 of the *Dmd* gene in BALB/c mice. Analysis of these mice at 3, 6 and 12 months of age confirmed the expected dystrophic pathology in the limb muscles and diaphragm as well as compromised trabecular bone development. This new murine model is therefore a useful addition to the preclinical model toolbox for the development and testing of novel therapies for DMD.

A dystrophic muscle phenotype such as that in *mdx* mice was confirmed, with evidence for limb muscle hypertrophy, centrally nucleated and variably sized muscle fibers, increased fibrosis, and membrane permeability. Limb muscle hypertrophy has been extensively reported in the C57BL/10ScSn *mdx* mouse and is generally attributed to a robust regenerative response (Coley et al., 2016; Duddy et al., 2015). This phenotype is not typically observed in the *mdx*/*utrn*^−/−^ mouse (Deconinck et al., 1997; Ham et al., 2019) or D2.*mdx* mouse (Coley et al., 2016), which usually exhibit more of an atrophic phenotype. Interestingly, the C.*mdx*62 mouse presents with a phenotype intermediate to these models with hypertrophy of the TA and soleus muscles, variable changes in mass of the EDL and no change in the gastrocnemius. The mechanism responsible for these muscle-specific changes remains to be determined and should be explored in future studies using genomic and proteomic profiling to detect specific differences between muscle groups.

Absolute force production was maintained in the TA muscles of C.*mdx*62 mice relative to that in WT mice at 3 and 12 months of age and increased at 6 months of age. Like in C57BL/10ScSn *mdx* mice, this likely resulted from increased muscle mass as normalized force was lower relative to that in WT mice (Lynch et al., 2001; Sacco et al., 1992). Interestingly, normalized force was not different between WT and C.*mdx*62 mice at 6 months of age, but this was likely due to reduced force in the WT mice as normalized force was stable across the 3-, 6- and 12-month age groups in the C.*mdx*62 mice but changed with age in the WT mice. As the WT mice used in this study were purchased separately and, therefore, did not exactly match the genetics of the C.*mdx*62 mice, future studies should incorporate littermate controls to eliminate this confounding factor.

As reported previously in preclinical models and in patients with DMD (Swiderski and Lynch, 2021), the pathology was more significant in the diaphragm than in limb muscles. This was confirmed by significant ectopic myofiber calcification in the diaphragm of C.*mdx*62 mice at all ages relative to that in WT mice, similar to that reported previously in *mdx^βgeo^* and D2.*mdx* mice (Hammers et al., 2020; Mazala et al., 2020; van Putten et al., 2019; Young et al., 2020). In the diaphragm muscles of D2.*mdx* mice, ectopic myofiber calcification has been attributed to a polymorphism in the *Ltbp4* gene from crossing the *mdx* mouse onto the DBA/2J strain (Coley et al., 2016). BALB/c mice exhibit dystrophic cardiac calcinosis of the epicardia and are more susceptible to developing ectopic calcification (Eaton et al., 1978). The increased ectopic myofiber calcification in the diaphragm of C.*mdx*62 mice may therefore be attributed to the BALB/c strain background. Alternatively, the worsened phenotype may arise from the loss of additional dystrophin isoforms, as was suggested in the *mdx*^βgeo^ mouse, which lacks all dystrophin isoforms (Young et al., 2020), but this remains to be confirmed.

The characterization of the C.*mdx*62 mouse presented here demonstrates the expected dystrophic pathology in the limb muscles and diaphragm. However, respiratory and cardiac failure are the two most common causes of death in DMD (Mercuri et al., 2019), and these are less well modelled in mice. Similar to DBA/2J mice, BALB/c mice are prone to dystrophic cardiac calcinosis (Glass et al., 2013). In fact, two of the five 12-month-old WT BALB/c mice in the present study exhibited significant cardiac calcification. Interestingly, hearts from 12-month-old C.*mdx*62 mice studied here exhibited signs of fibrosis at dissection, but with the exception of a main effect for increased *Runx2* expression, there was no evidence of ectopic cardiac calcification in these mice. The presence of a cardiac phenotype in WT BALB/c mice may influence the interpretation of any phenotype in the C.*mdx*62 mouse. Our preliminary characterization suggests that C.*mdx*62 mice have a cardiac phenotype from 6 months of age, indicated by a reduced heart mass and SERCA dysfunction. Reduced heart mass has similarly been observed in *mdx*/*utrn*^−/−^ mice (Kennedy et al., 2016) and increased heart mass has been reported to occur at least transiently in D2.*mdx* mice, with no change in C57BL/10ScSn *mdx* mice up to approximately 6 months of age (Kennedy et al., 2018). Future studies examining cardiac histology, cardiac function and respiratory capacity are required to determine whether the C.*mdx*62 model develops a cardiorespiratory phenotype and how this relates to the spectrum of phenotypes in existing models.

Poor bone health is a common yet serious complication of pediatric neuromuscular diseases, including, but not limited to, spinal muscular atrophy (Vai et al., 2015), cerebral palsy (Modlesky et al., 2009), myelomeningocele (Chadwick et al., 2018) and DMD. Reduced mobility and other systemic factors lead to a common presentation of abnormal bone development and architecture, with low bone mass and mineral density (Veilleux and Rauch, 2017). In DMD, up to 20-25% of patients experience a long bone fracture (McDonald et al., 2002), with those occurring in the lower extremities often leading to a permanent loss of ambulation (Larson and Henderson, 2000). Furthermore, the osteotoxic effects of chronic glucocorticoid therapy significantly increases the prevalence of vertebral fractures (King et al., 2007; Ward et al., 2018). Although the pathophysiological mechanisms driving poor bone health are unclear, it remains important to understand and identify effective avenues to improve the quality of life for patients. We report here that the C.*mdx*62 mouse model exhibits compromised trabecular bone development and accrual during the later stages of skeletal development and maturation (i.e. after 3 months of age). This is a likely consequence of the increased inflammation and dystrophic phenotype of the skeletal muscle and is consistent with previous reports in *mdx* mice (Nakagaki et al., 2011; Novotny et al., 2011; Ponzetti et al., 2022; Rufo et al., 2011; Wada et al., 2019). Interestingly, the age-related loss of trabecular bone was attenuated in older C.*mdx*62 mice and, although interesting, the underlying mechanism for this remains unclear and warrants further investigation.

Unlike our observations of the trabecular bone, the C.*mdx*62 mouse model did not exhibit a cortical bone phenotype that is reminiscent of that observed in patients with DMD. The C.*mdx*62 mouse exhibits age-related increases in cortical bone mass, diameter, thickness and tissue mineral density, relative to those of their WT counterpart. The effect of muscular dystrophy on cortical bone has been less clear, with some studies reporting *mdx* mice having slender, thinner and weaker cortical bone (Anderson et al., 1993; Nakagaki et al., 2011; Novotny et al., 2011; Rufo et al., 2011; Wada et al., 2019), whereas others have reported no differences in cortical thickness or diameter (Wood et al., 2020) as well as increased bone mineral density (Montgomery et al., 2005). These inconsistencies could be due to the different ages, sex and region of long bone analyzed, and it remains difficult to define the pathophysiological mechanism(s) underlying the influence of dystrophic muscle on the skeleton. Although our data suggests that these structural (extrinsic) changes to cortical bone would improve bone strength, this remains to be confirmed through mechanical testing. Nevertheless, we propose the potential use of the C.*mdx*62 mouse as a tool to identify modifiable and/or protective factors that may be absent in *mdx* mice on the C57BL/10 background strain.

We also report that bone retains the ability to structurally adapt to the adjacent skeletal musculature, independent of the dystrophic phenotype of C.*mdx*62 mice. We propose that the lengthening of the tibial crest in C.*mdx*62 mice is the direct result of the larger TA muscle. We previously reported this phenomenon in healthy mice in response to follistatin (FST)-induced muscle hypertrophy (Chan et al., 2021) and in dystrophic mice after muscle remodeling (Chan et al., 2023). These findings are akin to a previous study reporting expansion of the third trochanter in response in *mdx* mice (Montgomery et al., 2005) and in myostatin (*Mstn*)-null mice (Hamrick et al., 2000; Hamrick et al., 2002). We propose that the deficit in trabecular bone development is predominantly driven by the dystrophic muscle phenotype, whereas the differences in cortical bone structure are likely an adaptive response to accommodate the larger, adjacent musculature. We speculate there are two possibilities that could account for the differential phenotypes between trabecular and cortical bone. Firstly, the rate of bone modeling and remodeling differ between these two compartments, with the trabecular bone being more susceptible to imbalances in formation and resorption owing to its higher turnover rate and larger surface area-to-volume ratio. Secondly, dystrophin may play an intrinsic role in bone-resident cell types. Although this has not been confirmed, it remains interesting and worthy of further investigation. Collectively, our data add merit to the C.*mdx*62 mouse being a unique and valuable model for investigating common and divergent mechanisms across mouse strains, as well as being a suitable model for studying the rapid loss of trabecular bone as a consequence of glucocorticoid therapy (Tung et al., 2021).

CRISPR/Cas9 has been increasingly used to generate murine DMD models with mutations in specific regions. The advent of CRISPR/Cas9 technology provided a simpler and less time-consuming approach for generating novel models with mutations in specific regions for targeted study, with some mimicking variants in patients or humanizing the mouse gene, for preclinical evaluation of targeted exon skipping and/or gene-editing therapies (Amoasii et al., 2017; Kim et al., 2017; Koo et al., 2018; Maino et al., 2021; Min et al., 2019; Min et al., 2020; Rok et al., 2023; Ryu et al., 2018; Wong et al., 2020; Zhang et al., 2020). The majority of these models have been generated in C57BL/6J mice (or in strains crossed to C57BL/6J to generate a mixed background), with only a few examples in other backgrounds, including an exon 51 deletion in C57BL/6N mice (Chemello et al., 2020), and mouse models expressing mutations in the humanized *dmd* gene in C57BL/10, DBA/2J, or C57BL/6J mice (Kenjo et al., 2021; Pickar-Oliver et al., 2021; Young et al., 2017). Similar to the C.*mdx*62 mouse, all these models exhibit an expected dystrophic pathology for mutations resulting in the loss of expression of the full-length dystrophin protein. C.*mdx*62 represents a novel mouse model with both a different mutation location and background strain.

The 8 bp deletion in exon 62 in these mice should affect expression of all dystrophin isoforms, with the exception of Dp71 and Dp40, for which the promoter is located in intron 62 (Hugnot et al., 1992; Lederfein et al., 1993; Rapaport et al., 1992), just downstream of this mutation. Analysis of sciatic nerve protein extracts confirmed the loss of Dp116 expression in these mice. Loss of Dp260 and Dp140 could not be confirmed due to the inability to detect these isoforms in the brains of BALB/c WT mice, but it can be implied based on the locations of the promoters for these isoforms. There is potential for non-muscle phenotypes in these mice, such as those reported previously in *mdx*52 mice (Barboni et al., 2021; Saoudi et al., 2021). Importantly, this is the first mouse model with a mutation at exon 62. Given that mutations in this region result in the DMD phenotype, with mutations in exon 62 accounting for 0.88% of reported variants in the Leiden Open Variation database (Unique variants in the DMD gene, Global Variome shared LOVD; https://databases.lovd.nl/shared/variants/DMD/unique), this mouse may prove to be an important tool for testing exon-skipping strategies relevant to exon 62 gene mutations.

Although multiple murine models of DMD have been generated using various strategies in the C57BL/6, C57BL/10 and DBA/2J mouse strains, this is the first model, to our knowledge, characterized in the BALB/c mouse strain. As the immunological phenotype of BALB/c mice varies relative to that of C57BL/6 mice, with stronger Th2 and humoral responses (Watanabe et al., 2004), it is likely that mice on a BALB/c background respond differently to therapeutic interventions. Individual immune responses vary greatly in the human population, and immune response variability to some therapies, particularly viral-mediated interventions, has led to a shortfall in successful translation to the clinic and may limit the dose that can be safely administered to patients. Furthermore, the skeletal muscles of BALB/c mice have been reported to have a reduced regenerative capacity after femoral artery ligation compared to those of C57BL/6 mice (Tu et al., 2022) and, thus, it may be expected that the dystrophic progression varies between different mouse strains. Preclinical analysis of interventions in DMD mouse models across multiple background strains with varying immune profiles and regenerative capacity, are perhaps more closely representative of variations in patient populations and may improve translation to the clinic.

In conclusion, C.*mdx*62 mice represent the first dystrophin-deficient murine model on a pure BALB/c background with an 8 bp deletion in exon 62, which exhibit a dystrophic pathology in limb muscles and diaphragm and a bone phenotype up to 12 months of age. Although continued studies into the pathology and mechanisms underlying dystrophic progression will prove valuable in understanding this model, the variation in background strain and mutation location makes the C.*mdx*62 mouse an important addition to the preclinical toolbox for DMD research.

## MATERIALS AND METHODS

### Experimental animals

All experiments were approved by the Animal Ethics Committee of The University of Melbourne and conducted in accordance with the Australian code for the care and use of animals for scientific purposes as stipulated by the National Health and Medical Research Council (Australia). Male BALB/c WT mice were obtained from the Animal Resources Centre (Canning Vale, Western Australia) and BALB/c C.*mdx*62 mice were bred in the Biological Research Facility at the University of Melbourne. All mice were housed in the Biological Research Facility under a 12 h:12 h light-dark cycle, with water and standard laboratory chow available *ad libitum*.

### CRISPR-mediated mutagenesis of dystrophin

C.*mdx*62 mice were created in collaboration with the Melbourne Advanced Genome Editing Centre (MAGEC) facility at the Walter and Eliza Hall Institute (Melbourne, Australia). To generate mice, 20 ng/μl of Cas9 mRNA, 10 ng/μl of sgRNA (5ʹ-GGGCACTTTGTTTGGTGAGA-3ʹ) and 40 ng/μl of oligonucleotide donor (5ʹ-tttcttttctttttcctttttttctttttgcagCTTCAGTTCAGGGTCCCTGGGAGAGAGCtATCGAGCCAAACAAAGTGCCCTACTATATCAAgtaagtcaaaagcatttatgtacctgatctgtat-3ʹ) were injected into the cytoplasm of fertilized one-cell-stage embryos derived from WT BALB/c breeders. Viable founder mice were identified by next-generation sequencing. Targeted animals were backcrossed onto BALB/c mice for two generations to eliminate potential sgRNA off-target hits. Founder mice were backcrossed to WT BALB/c mice for two generations and tail snips were genotyped through Transnetyx (Cordova, TN, USA) to identify the presence or absence of the 8 bp deletion. Once backcrossed, mice were interbred to generate a homozygous colony. These mice have been cryopreserved and are available for use via the Australian Phenome Bank (strain ID: 9150; Australian Phenome Bank; https://www.phenomebank.org).

### Muscle function

At 3, 6 or 12 months of age, mice were anesthetized with sodium pentobarbital (Nembutal; 60 mg/kg, Sigma-Aldrich, Castle Hill, New South Wales, Australia) via intraperitoneal injection and contractile properties of the TA muscles assessed *in situ* with an intact nerve and blood supply, as described previously (Gehrig et al., 2010; Hardee et al., 2021; Lynch, 2004). This included assessments of isometric twitch and tetanic contractile characteristics and the frequency-force relationship, as outlined in the TREAT-NMD SOP (DMD_M.2.2.005, https://www.treat-nmd.org/wp-content/uploads/2023/07/MDX-DMD_M.2.2.005.pdf), followed by an assessment of the muscle susceptibility to contraction-mediated injury, based on the cumulative deficit in isometric force production after repeated lengthening contractions. Isolated muscles were maximally activated to produce isometric force and then stretched to perform an eccentric contraction (at a velocity of 2 fiber lengths (L_f_)/s) at progressively increasing magnitudes of stretch. Maximum isometric force was determined before each eccentric contraction. The muscles were set at an increasing resting length to a maximum of 30% L_f_ and stimulated at at 120 Hz. At the conclusion of these measurements, the muscles of the left hindlimb (including the TA, EDL, soleus and quadriceps) as well as the epididymal fat, spleen, liver, diaphragm and heart were excised, trimmed of tendon and any non-muscle tissue, blotted once on filter paper and weighed on an analytical balance. The EDL, soleus, quadriceps, epididymal fat, liver and a piece of diaphragm muscle were snap frozen for later analyses. The TA and a second piece of diaphragm muscle were mounted in optimal cutting temperature (OCT) compound and frozen in thawing isopentane for later histochemical analyses. Mice were killed as a consequence of diaphragm and heart excision while they were anesthetized deeply.

### Serum analysis

Whole blood was collected from mice (*n*=5-9/group) into Eppendorf tubes at endpoint via cardiac puncture. To isolate serum, tubes were left incubated for 30 min at room temperature for 30 min, then centrifuged at 5000 ***g*** for 10 min at 4°C and the serum was removed to fresh Eppendorf tubes and stored at −80°C. As serum creatine kinase levels from dystrophic mice are in the range of 1339-55620 U/l, with a mean±s.d. of 18,532±14,577 U/l (TREAT-NMD SOP MD_M2.2.001 Serum Creatine Kinase analysis in mouse models of muscular dystrophy, https://www.treat-nmd.org/wp-content/uploads/2023/07/MDX-MD_M.2.2.001.pdf), which is outside the measurable range of the Equine Profile Plus rotor (0-14,000 U/l), it was not possible to accurately assess these levels in the C.*mdx*62 mouse. Therefore, levels of serum AST were used as a biomarker of DMD muscle pathology and measured on a VetScan VS2 chemistry analyzer (Abaxis, Union City, CA, USA) using the Equine Profile Plus rotor according to the manufacturer's protocol.

### Western blotting

For protein analyses, tissues were homogenized in ice-cold buffer (10 mM Tris-HCl, pH 7.4, 100 mM NaCl, 1 mM EDTA, 1 mM EGTA, 1 mM NaF, 1% Triton X-100, 10% glycerol, 0.1% SDS, 20 mM Na_4_P_2_O_7_, 0.5 mM Na_3_VO_4_, 0.5% sodium deoxycholate, 0.1 mM PMSF, and protease and phosphatase inhibitors; all from Sigma-Aldrich). Samples were centrifuged at 10,000 ***g*** for 5 min at 4°C and the resulting supernatant analyzed for total protein content (DC Protein Assay; Bio-Rad Laboratories), with bovine serum albumin (BSA) standards. Samples were normalized to 2 μg/μl in homogenizing buffer containing 4× Laemmeli sample buffer (0.25 M Tris-HCl, pH 6.8, 6% SDS, 40% glycerol, 0.04% bromophenol blue, 16% dithiothreitol) and heated for 3 min at 95°C. Lysates were run on 4-15% Criterion TGX Stain-Free gels (Bio-Rad Laboratories) at a constant voltage of 100 V and transferred to PVDF membranes. Membranes were blocked for 1 h at room temperature (RT) in TBS containing 0.1% Tween 20 (TBST) and 5% BSA and incubated with primary antibodies diluted in 5% BSA/TBST overnight at 4°C. The following primary antibodies were used: mouse anti-dystrophin [1:1000; MANEX1011B(1C7); Developmental Studies Hybridoma Bank], mouse anti-β-dystroglycan (1:500; B-DG-CE; Leica Biosystems), mouse anti-utrophin (1:1000; 610896; BD Biosciences), mouse anti-dystrophin (1:1000; MANDRA 7A10, D8043; Sigma-Aldrich), rabbit anti-phospho-phospholamban (Ser16) (1:1000; 07-052; Merck Millipore) and goat anti-phospholamban (1:1000; sc21923; Santa Cruz Biotechnology). The following day, membranes were washed in TBST and incubated for 1 h at RT in horseradish peroxidase (HRP)-conjugated sheep anti-mouse IgG (NA931; Cytiva Life Sciences), donkey anti-goat IgG (ab97110; Abcam) or donkey anti-rabbit IgG (NA934 V; Cytiva Life Sciences) at 1:5000 dilution in 5% BSA/TBST. After washing in TBST, membranes were treated with enhanced chemiluminescence (Immobilon Forte Western HRP substrate; EMD Millipore, Hayward, CA, USA). Samples were evaluated by integrated densitometry using a ChemiDoc XRS machine and Image Lab software (Bio-Rad Laboratories). To confirm equal loading between lanes, stain-free blots were imaged on the ChemiDoc according to manufacturer's instructions. Full western blots are shown in Fig. S7.

### Histology

Serial sections (8 μm) of TA or diaphragm muscles were cut transversely using a refrigerated (−20°C) cryostat (CTI Cryostat; IEC, Needham Heights, MA, USA) and stained with Hematoxylin and Eosin to assess overall muscle structure or with Van Gieson's stain to determine collagen infiltration as an indicator of fibrosis, as described previously (Gehrig et al., 2010). Digital images of stained sections were obtained using an upright microscope with a camera (Axio Imager, Carl Zeiss, Wrek, Göttingen, Germany), controlled by AxioVision AC software (AxioVision AC Rel. 4.8, Carl Zeiss Imaging Solutions, Wrek, Göttingen, Germany). Images were quantified for fibrosis content using FIJI/ImageJ software (FIJI v2.5.0).

### Immunofluorescence

For assessment of muscle fiber type and oxidative capacity, transverse sections (8 μm) were reacted with SDH, as described previously (Blanco et al., 1988), rinsed 3× dH2O, dehydrated through 30%, 60%, and 90% acetone, and air dried for 15 min. Sections were rinsed with 0.1% PBS + 0.1% Tween-20 for 10 min and incubated in primary antibodies including rabbit-anti-laminin IgG (L9393; Sigma-Aldrich), mouse-anti-Myosin Heavy Chain (MHC) IIa IgG1 (SC-71) and mouse-anti-MHC IIb IgM (BF-F3; both developed by Stefano Schiaffino, University of Padova (Padua, Italy), obtained from the Developmental Studies Hybridoma Bank), diluted at 1:25, 1:25, and 1:10 respectively in 5% normal goat serum/0.05% PBST for 2 h at RT. Sections were washed three times for 5 min in PBS and incubated in secondary antibodies including Alexa Fluor 647-conjugated goat-anti-mouse IgG1 (A21240; Thermo Fisher Scientific, Waltham, MA, USA), Alexa-Fluor 488-conjugated goat-anti-mouse IgM (A21042; Thermo Fisher Scientific), and Alexa-Fluor 555-conjugated goat-anti-rabbit IgG (A21428; Thermo Fisher Scientific) diluted at 1:100, 1:250, and 1:250 respectively in 5% normal goat serum/0.05% PBST for 1 h at RT. Sections were washed three times for 5 min in PBS, and mounted onto coverslips with Mowiol. For detection of syntrophin and sarcoglycan, frozen sections were thawed at RT and incubated for 1 h at RT in either mouse anti-α-sarcoglycan (NCL-L-a-SARC; Leica Biosystems) or mouse anti-syntrophin (SAB4200213; Sigma-Aldrich) at 1:100 dilution in 10% normal goat serum in TBS. Sections were rinsed in TBS two times for 5 min, incubated for 1 h at RT in Alexa Fluor 647-conjugated goat anti-mouse IgG1 secondary antibody (A21240; Invitrogen) at 1:400 dilution in 10% normal goat serum in TBS, incubated with DAPI (1:1000 in TBS) for 10 min at RT, rinsed in TBS two times for 5 min, then dipped in PBS.

For detection of utrophin and the acetylcholine receptor (AChR), sections were fixed in ice-cold acetone for 2 min at RT, rinsed in PBS three times, then blocked in 5% normal goat serum in 1% BSA for 1 h at RT. Sections were incubated at 4°C in mouse anti-utrophin (sc-33700; Santa Cruz Technologies; 1:50) and mouse anti-AChR (610989; BD Biosciences; 1:50) in 1% BSA/PBS. The next day, sections were rinsed in 0.1% Triton X-100/PBS three times for 5 min, incubated with Alexa Fluor 647-conjugated goat anti-mouse IgG1 (A21240; Invitrogen) and Alexa Fluor 488-conjugated goat anti-mouse IgG2a (A21131; Invitrogen) at 1:400 dilution in 1% BSA/PBS for 1 h at RT, stained with DAPI (1:5000 in 0.1% Triton X-100/PBS) for 10 min at RT, and rinsed in 0.1% Triton X-100/PBS three times for 5 min.

For detection of dystrophin and β-dystroglycan, sections were fixed in 4% paraformaldehyde for 15 min at RT, rinsed in in 0.1% Triton X-100/PBS two times for 5 min, then subjected to heat-activated antigen retrieval in citrate buffer (pH 6.0, Sigma-Aldrich) in a pressure cooker for 10 min. Once cooled, sections were rinsed in 0.1% Triton X-100/PBS two times for 5 min, blocked in 10% normal goat serum and 3% BSA for 45 min at RT, and incubated at 4°C in mouse anti-dystrophin (MANDRA1 7A10, sc-47760; Santa Cruz Technologies; 1:100) and mouse anti-β-dystroglycan (NCL-bDG; Leica Biosystems; 1:50) in 3% BSA in 0.1% Triton X-100/PBS. The next day, sections were rinsed in PBS containing 0.1% Tween 20 three times for 10 min, incubated with Alexa Fluor 647-conjugated goat anti-mouse IgG1 (A21240; Invitrogen; 1:200) and Alexa Fluor 555-conjugated goat anti-mouse IgG2a (A21137; Invitrogen; 1:400) in 3% BSA/TBST for 1 h at RT, stained with DAPI (1:5000 in 0.1% Triton X-100/PBS) for 10 min at RT, and rinsed with 0.1% Triton X-100/PBS three times for 10 min.

For detection of laminin and IgG, sections were fixed in 4% paraformaldehyde for 10 min at RT, rinsed in PBS, washed in 0.1% Triton X-100/PBS (two times for 5 min), blocked in 10% normal goat serum in 2% BSA in 0.1% Triton X-100/PBS for 1 h at RT, and incubated for 1 h at RT in rabbit anti-laminin antibody (L9393; Sigma-Aldrich; 1:25) in 2% BSA in 0.1% Triton X-100/PBS. Sections were washed three times for 5 min with 0.1% Triton X-100/PBS, then incubated for 2 h at RT in Alexa Fluor 555-conjugated goat anti-rabbit IgG (A21428; Invitrogen) and Alexa Fluor 488-conjugated goat anti-mouse IgG (H+L, A11029; Invitrogen) secondary antibodies at 1:250 dilution in 2% BSA in 0.1% Triton X-100/PBS. Sections were incubated with DAPI (1:5000 in in 0.1% Triton X-100/PBS) for 10 min at RT and rinsed in 0.1% Triton X-100/PBS three times for 5 min.

At the conclusion of staining, all sections were air dried and mounted with glass coverslips using Mowiol, then allowed to dry overnight prior to imaging. Digital images of stained sections were obtained using an upright microscope with a camera (Axio Imager), controlled by AxioVision AC software (AxioVision AC Rel. 4.8). Images were quantified for muscle fiber size, percentage of central nuclei or percentage of IgG-positive muscle fibers using FIJI/ImageJ software.

### Quantitative real-time PCR

Total RNA was extracted from the heart (*n*=5-9/genotype/timepoint) using TRIzol/chloroform, followed by the RNeasy Fibrous Tissue Mini Kit (Qiagen) as per the manufacturer's instructions. The concentration and quality of RNA samples were determined using a Nanodrop 2000 spectrophotometer (Thermo Fisher Scientific). Real-time reverse transcription PCR was performed as described previously (Murphy et al., 2019; Swiderski et al., 2016) using the following forward and reverse primer sequences: *F4/80*, 5′-CATCAGCCATGTGGGTACAG-3′ and 5′-CATCACTGCCTCCACTAGCA-3′; *TGFβ*, 5′-TGAGTGGCTGTCTTTTGACG-3′ and 5′-TCTCTGTGGAGCTGAAGCAA-3′; *Col1a1*, 5′-CACCCTCAAGAGCCTGAGTC-3′ and 5′-GTTCGGGCTGATGTACCAGT-3′; *Col3a1*, 5′-ACCAAAAGGTGATGCTGGAC-3′ and 5′-GACCTCGTGCTCCAGTTAGC-3′; *Col6a1*, 5′-CCCCATTGGACCTAAAGGAT-3′ and 5′-TCTCCCACTTCACCCTCATC-3′; *Runx2*, 5′-GCCTTCAAGGTTGTAGCCCT-3′ and 5′-GTTCTCATCATTCCCGGCCA-3′; *Ocn*, 5′-TTCTGCTCACTCTGCTGACC-3′ and 5′-GGGACTGAGGCTCCAAGGTA-3′; and *Alpl*, 5′-CAGGCCGCCTTCATAAGCA-3′ and 5′-AATTGACGTTCCGATCCTGC-3′. Gene expression was quantified using a cycle threshold (C_T_) method. Relative gene expression was calculated using the expression 2^−ΔCT^, normalized to total cDNA content as determined using the Quant-iT OliGreen ssDNA assay kit and Quant-iT OliGreen ssDNA reagent (Thermo Fisher Scientific).

### Micro-computed tomographic scanning and analysis

Tibiae were carefully excised and fixed in 4% paraformaldehyde for 24 h, rinsed in PBS and stored in PBS. Tibiae were scanned using the Skyscan 1276 (micro-computed tomography imager, Bruker, Kontich, Belgium) at 9 μm resolution, 0.25 mm aluminium filter, 56 kV voltage, 200 μA, 560 ms exposure time, and 0.4° step rotation with frame averaging of 2. Images were reconstructed and analyzed using NRecon (v1.7.4.6, Bruker), Dataviewer (v1.5.6.2, Bruker), CT Analyzer (CTAn; v1.18.8.0, Bruker) and CTVox (v3.3.0 r1403, Bruker). Tibial lengths were determined after scanning. Regions of interest were determined as described previously (Chan et al., 2021). The trabecular bone was assessed in the proximal region commencing at 3% of bone length from the growth plate and extended distally for a total of 13.5% (equivalent to 0.5-3 mm of the growth plate). For cortical bone, a region of interest beginning at 50% of bone length and extending distally for 2% (approximately 0.5 mm) was used for three-dimensional cortical bone analyses using CT Analyzer. Representative images were taken at the mid-diaphysis (50% of bone length) using a pseudodensity filter in CTVox. The length of the tibial crest was measured using a custom script written in FIJI/ImageJ using individual cross-sectional images spanning 15 to 40% of the tibial length, as described previously (Chan et al., 2023).

### Statistics

Data were analyzed between groups using either a two-tailed unpaired Student's *t*-test or a two-way ANOVA with, where appropriate, Bonferonni's post hoc multiple comparisons test used to detect significant differences between means. Where data were determined to be not normally distributed, a Mann–Whitney U test was used. A *P*-value less than 0.05 was considered statistically significant. All statistical analyses were carried out using Prism GraphPad 6 software (GraphPad Software, La Jolla, CA, USA). All values are presented as mean±standard error of mean (±s.e.m.).

## Supplementary Material

10.1242/dmm.050502_sup1 Supplementary information

## Data Availability

All relevant data can be found within the article and its supplementary information.
