# Supplementary material for: The BALB/c.mdx62 mouse exhibits a dystrophic muscle pathology and is a model of Duchenne muscular dystrophy
Source: Dis Model Mech. 2024 May 3;17(4):dmm050502. doi: 10.1242/dmm.050502 (PMC11095634; doi:10.1242/dmm.050502)
Supplement: Supplementary information [file dmm-17-050502-s1.pdf]

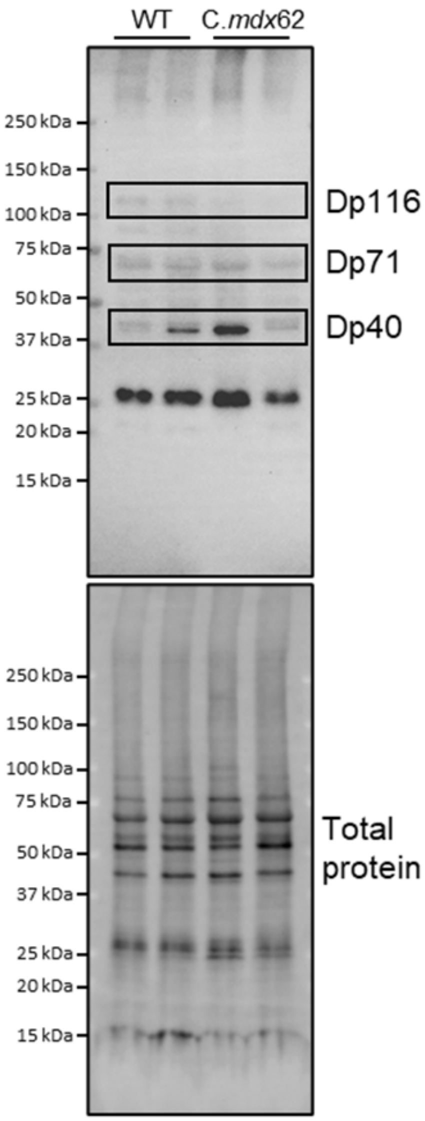

Supp. Fig. S1.

Fig. S1. C.*mdx*62 mice lack Dp116 but express Dp71 and Dp40. Sciatic nerve was dissected from 12-month-old BALB/c WT and C.*mdx*62 mice, protein extracted, and western immunoblotting undertaken to assess levels of Dp116, Dp71, and Dp40 dystrophin protein isoforms. Total protein was detected using the BioRad stain-free blot chemistry to confirm equal protein loading across samples.

Disease Models & Mechanisms • Supplementary information

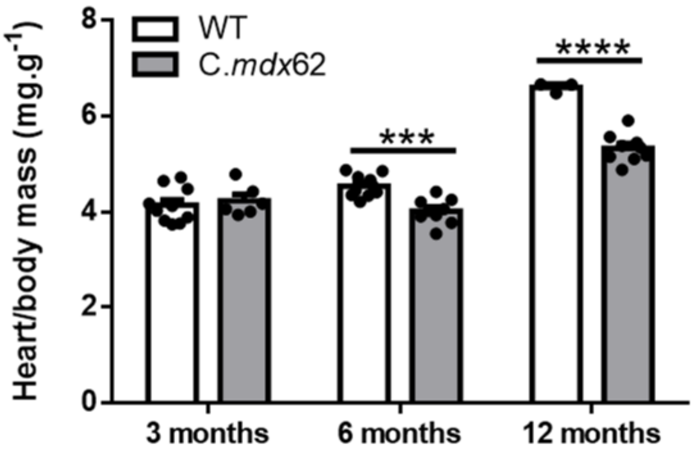

Supp. Fig. S2.

Fig. S2. C.*mdx*62 heart mass is reduced at 6 and 12 months of age. Data from Fig. 2F with the two abnormally large hearts removed from the 12 month old WT group confirming reduced normalize heart mass in the C.*mdx*62 mouse at 6 and 12 months of age. Data are presented as mean ± SEM. Statistical analysis using a two-way ANOVA with a post-hoc Bonferroni's test (with n = 3-10 mice/age/genotype). ***$P < 0.001$, ****$P < 0.0001$ relative to age-matched WT mice.

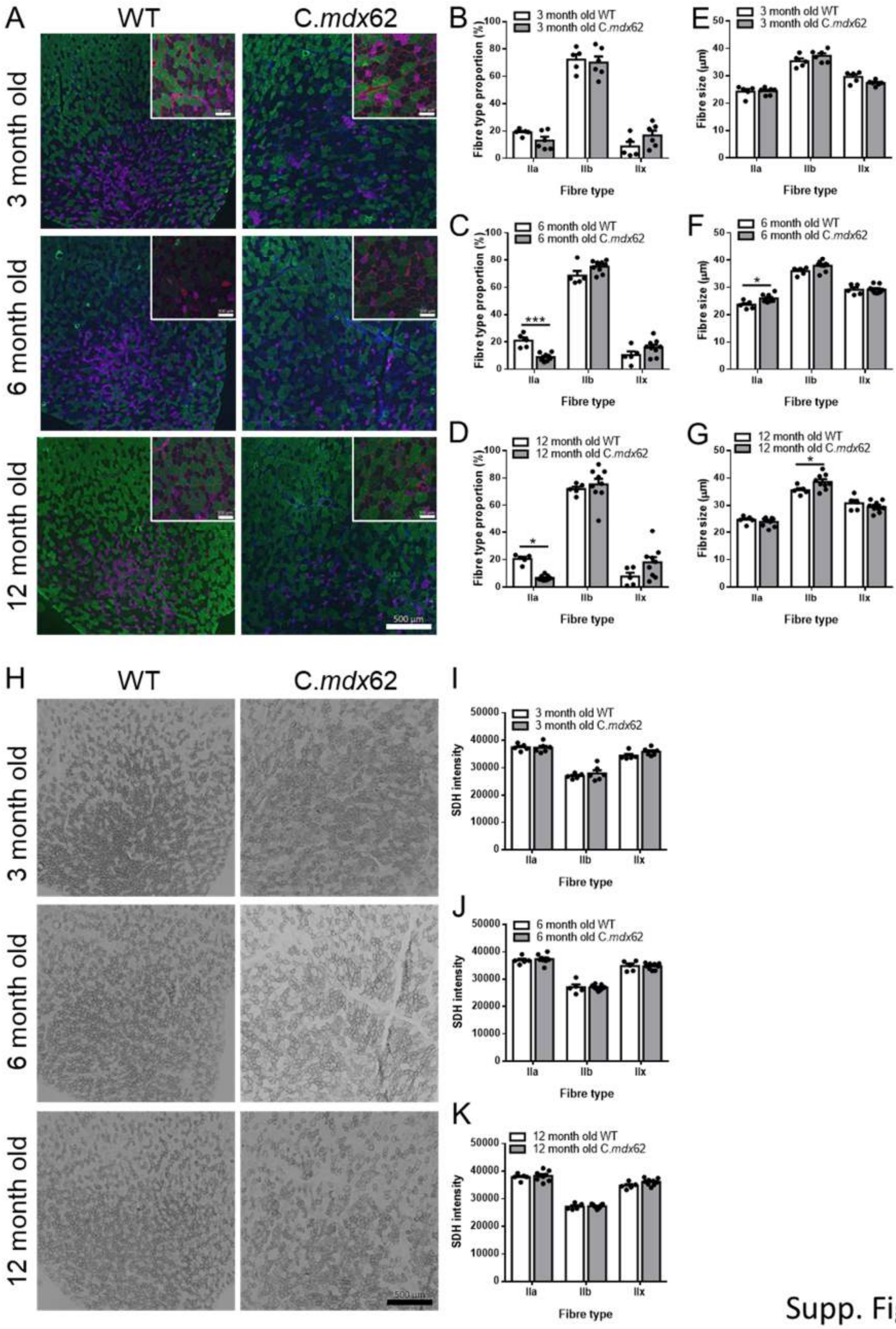
A
WT
C.mdx62
3 month old
6 month old
12 month old
500 µm
B
3 month old WT
3 month old C.mdx62
Fibre type proportion (%)
Fibre type
IIa
IIb
IIx
C
6 month old WT
6 month old C.mdx62
***
D
12 month old WT
12 month old C.mdx62
*
E
Fibre size (µm)
F
G
H
WT
C.mdx62
3 month old
6 month old
12 month old
500 µm
I
SDH intensity
J
K

Supp. Fig. S3.

Fig. S3. Muscle fiber type analysis in 3, 6, and 12 month old WT and C.*mdx*62 mice.

WT and C.*mdx*62 mutant mice were killed at 3, 6, and 12 months of age. TA muscles were excised, sectioned, and immunostained for type IIa (purple), type IIb (green), type IIx (black) muscle fibers, laminin (red), DAPI (nuclei, blue) (**A**). Stained sections were analyzed to determine the fiber type proportions (**B**-**D**) and fiber size (**E-G**). Sections were also immunoreacted for and for SDH (bright field) to assess oxidative capacity (**H**) and quantified for SDH intensity (**I**-**K**) of the different fiber types. Scale bar = 500 µm (100 µm for insets). Data are presented as mean ± SEM. Statistical analysis using a two-way ANOVA with a post-hoc Bonferroni's test (with n = 5-9 mice/age/genotype). **P* < 0.05, ****P* < 0.001 relative to age-matched WT mice.

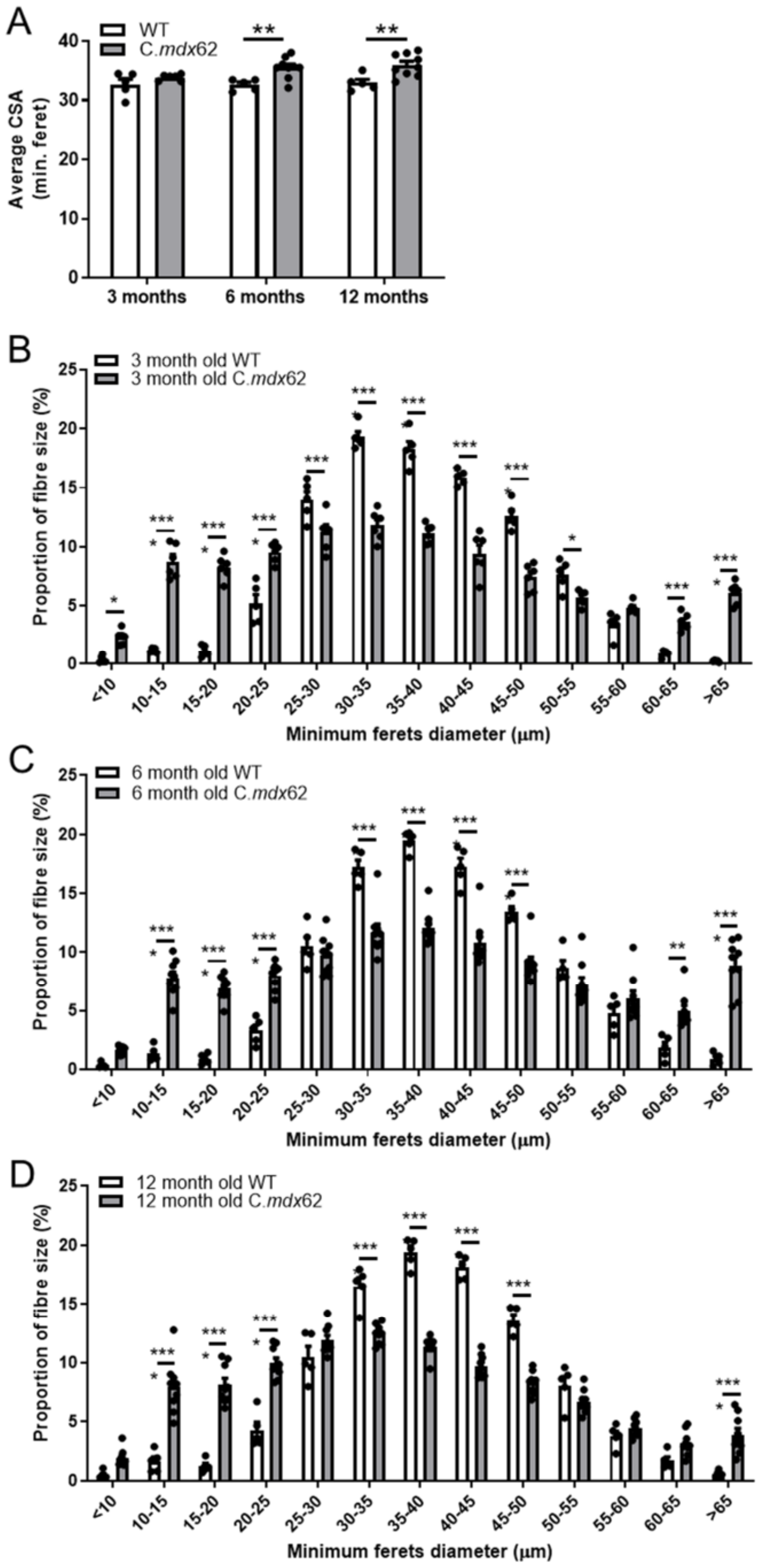

Supp. Fig. S4.

Disease Models & Mechanisms • Supplementary information

Fig. S4. C.*mdx*62 mice exhibit variable (and increased) muscle fiber size at 3, 6, and 12 months of age. WT and C.*mdx*62 mutant mice were killed at 3, 6, and 12 months of age. TA muscles were excised, sectioned, and immunostained for laminin. Stained sections were analyzed to determine average fiber size (CSA; **A**), as well as the size distribution of muscle fibers from 3 (**B**), 6 (**C**), and 12 (**D**) month old mice. Data are presented as mean ± SEM. Statistical analysis using a two-way ANOVA with a post-hoc Bonferroni's test (with n = 5-9 mice/age/genotype). **P* < 0.05, ***P* < 0.01, ****P* < 0.001, *****P* < 0.0001 relative to age-matched WT mice.

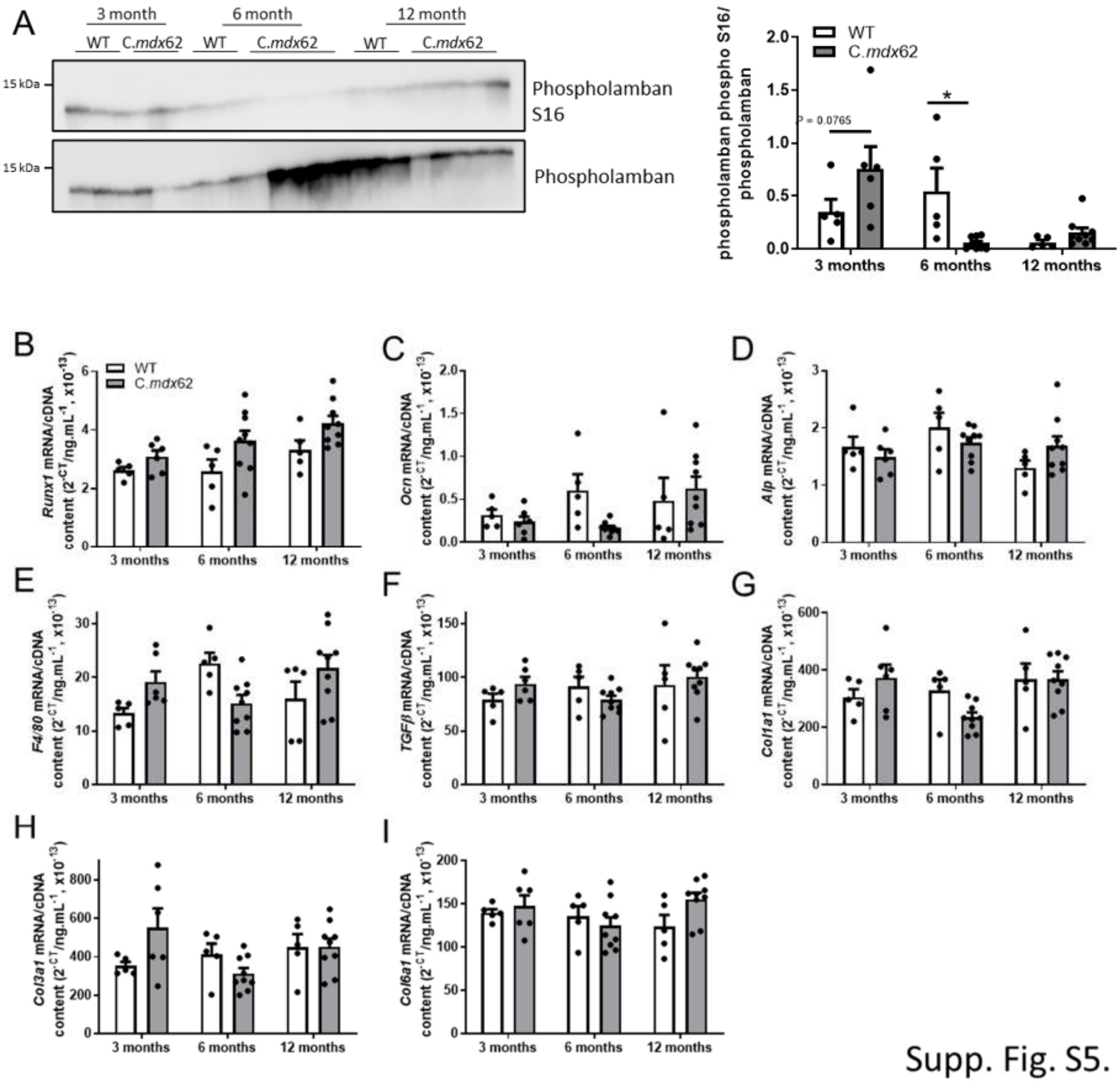

Fig. S5. C.*mdx*62 mice exhibit SERCA dysfunction in the heart. WT and C.*mdx*62 mutant mice were killed at 3, 6, and 12 months of age. Hearts were excised and protein and RNA extracted for analysis. (**A**) Protein was assessed for levels of phosphorylated and total phospholamban. cDNA was analyzed by qRT-PCR for mRNA expression of (**B**) *Runx1*, (**C**) *Ocn*, (**D**) *Alp*, (**E**) *F4/80*, (**F**) *Tgfβ*, (**G**) *Col1a1*, (**H**) *Col3a1*, and (**I**) *Col6a1*. Data are presented as mean ± SEM. Statistical analysis using a two-way ANOVA with a post-hoc Bonferroni's test (with n = 5-9 mice/age/genotype). *$P < 0.05$ relative to age-matched WT mice.

Disease Models & Mechanisms • Supplementary information

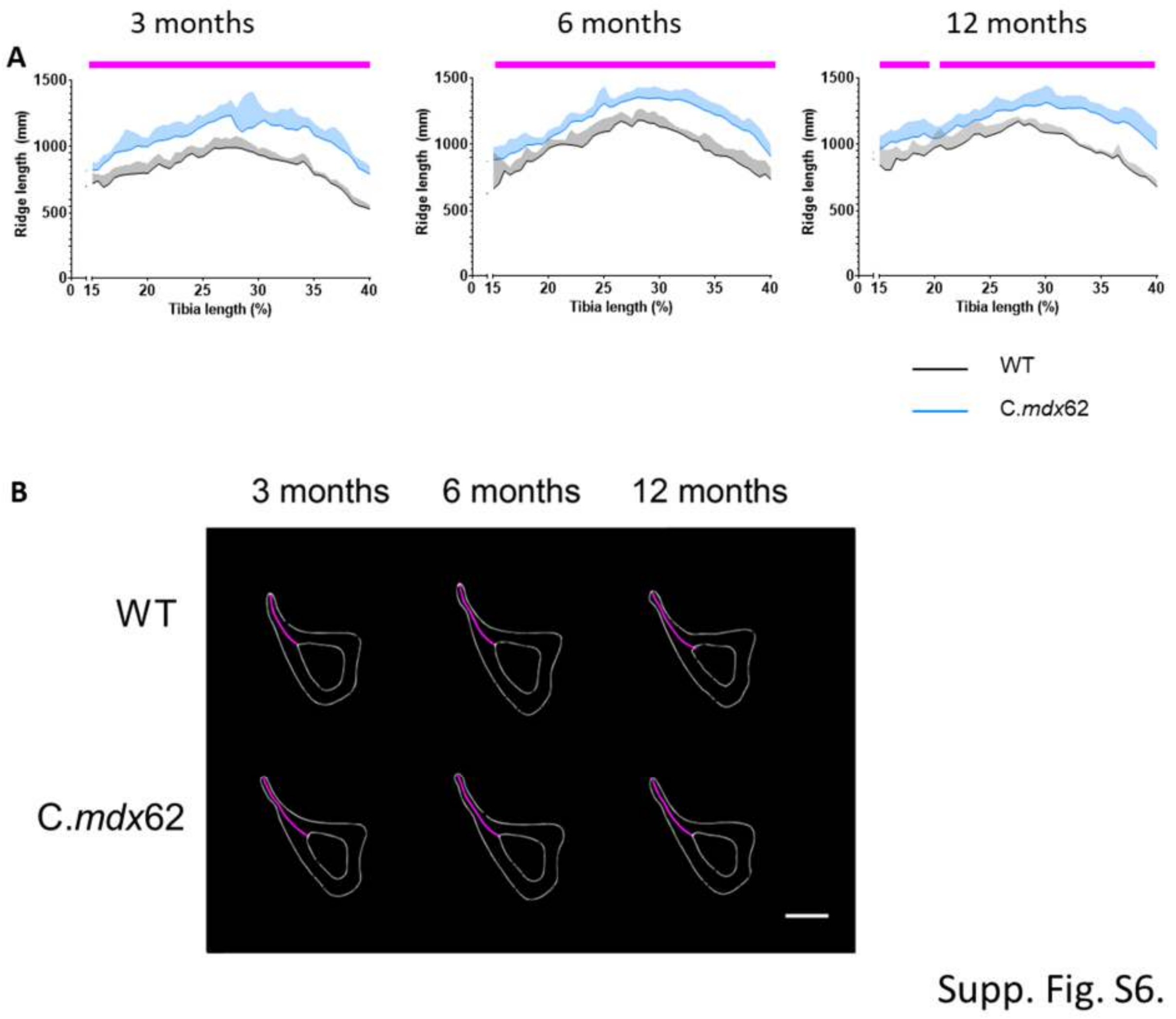

Fig. S6. Augmentation of the tibial crest during skeletal development in C.*mdx*62 mice. (**A**) Tibial crest length was measured using a customized macro plugin in ImageJ. Data + STD for BALB/c wildtype (WT) and C.*mdx*62 mice are represented in black and blue, respectively. Significant effects ($p<0.05$) are indicated by the solid pink bar above each graph, as determined by repeated measures two-way ANOVA with correction for multiple comparisons by the two-stage step-up method of Benjamini, Krieger, and Yekutieli (36). (**B**) Representative binary cross-sectional images are shown at 30% tibial length in WT and C.*mdx*62 mice at 3, 6 and 12 months of age. Periosteal (outer) and endocortical (inner) surfaces are outlined in white, and the tibial crest length is outlined in pink. Scale bar represents 1 mm.

Disease Models & Mechanisms • Supplementary information

Fig. S7. Full western immunoblots.

Fig. 1C

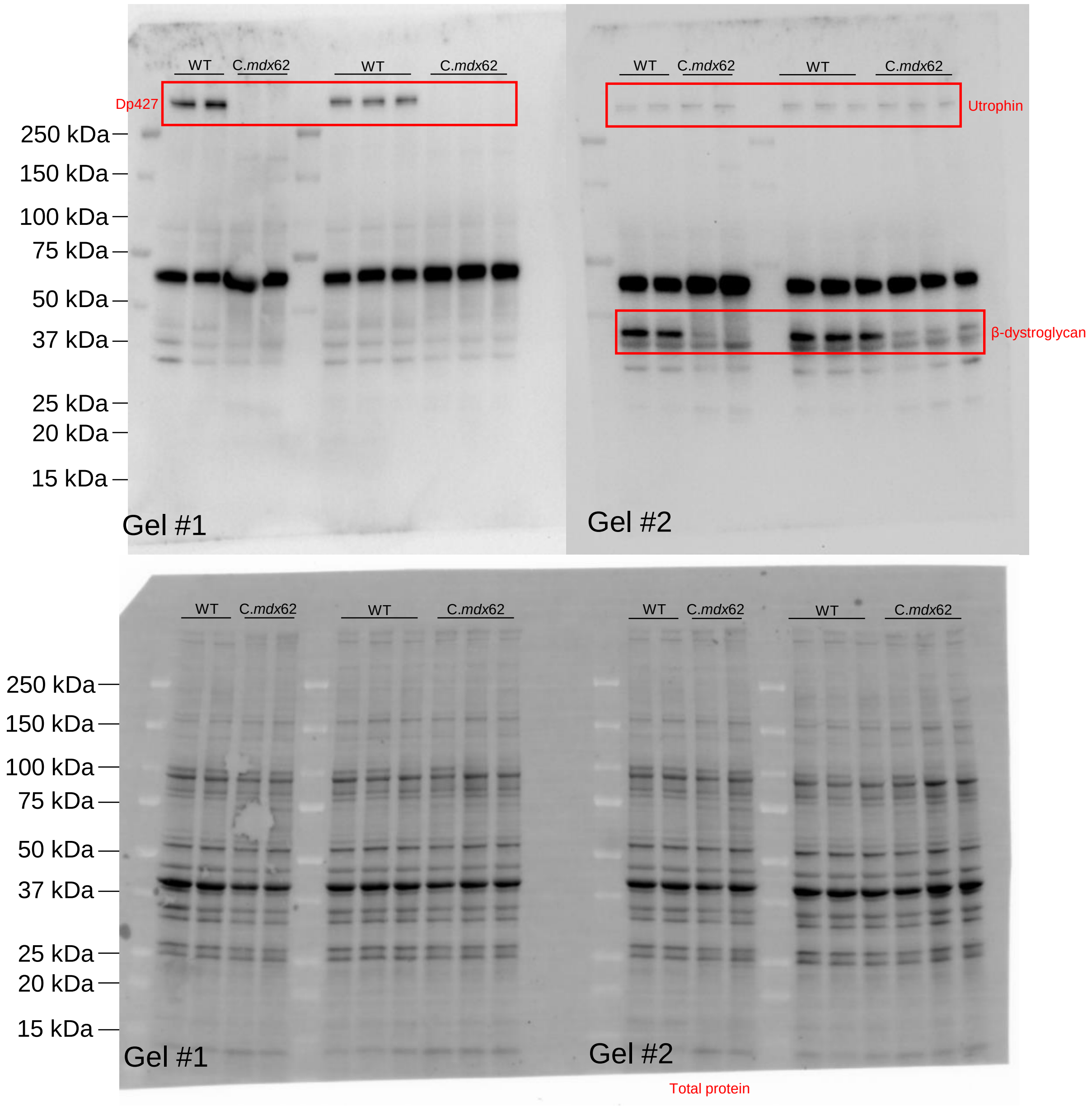

Disease Models & Mechanisms • Supplementary information

Fig. S1

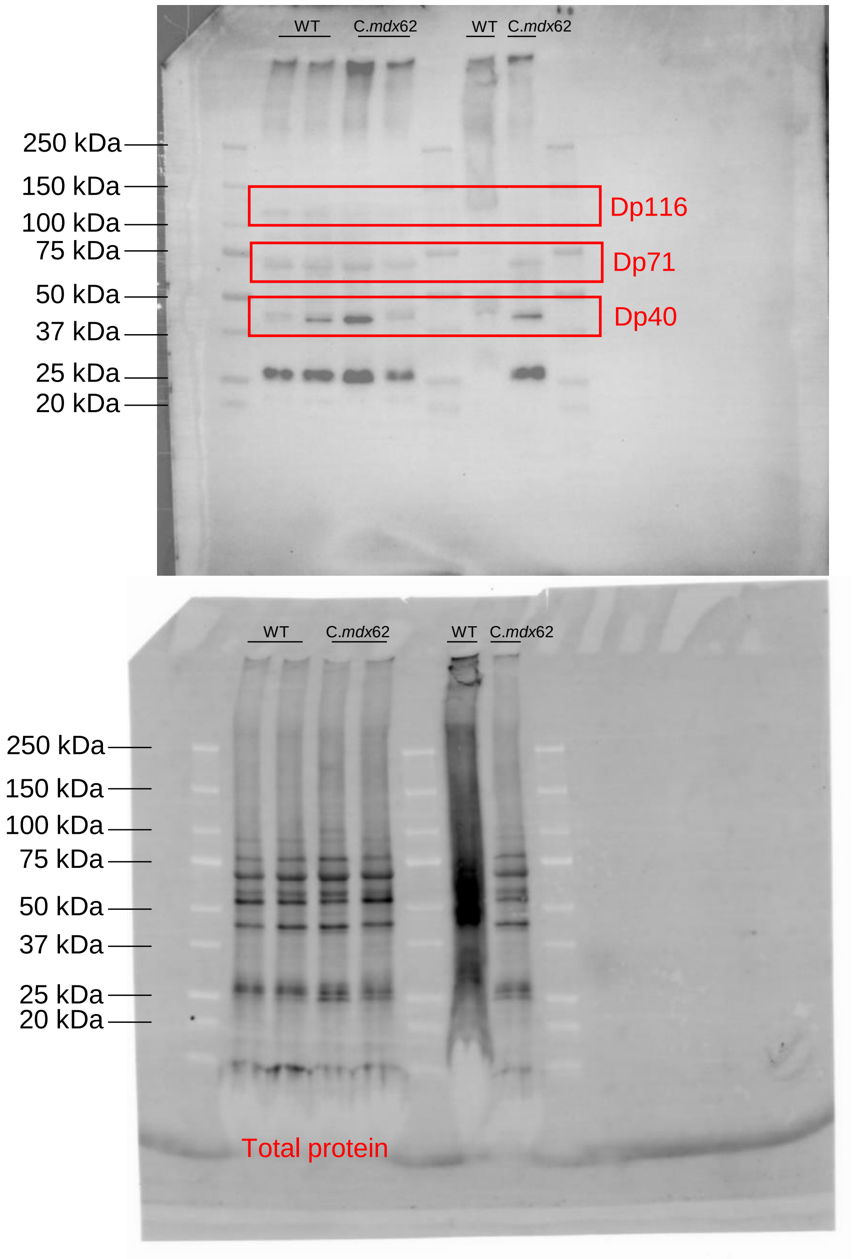

Disease Models & Mechanisms • Supplementary information

Fig. S4A

Set #1

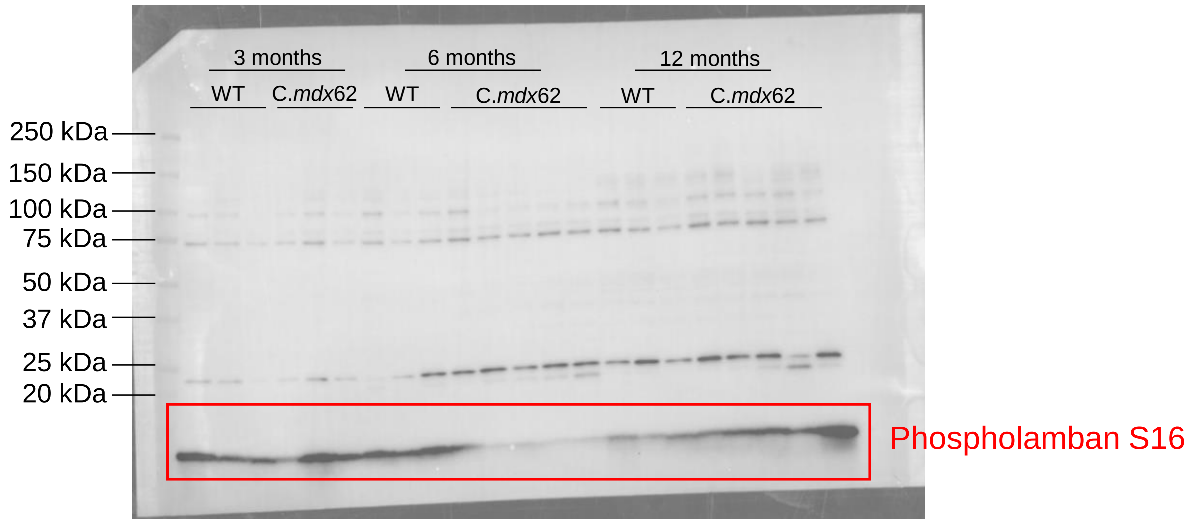

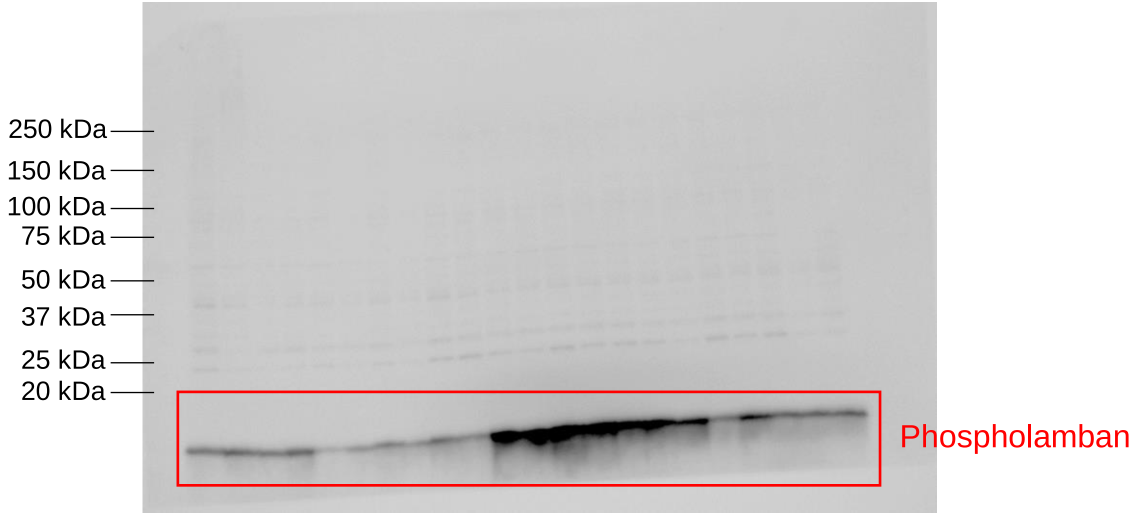

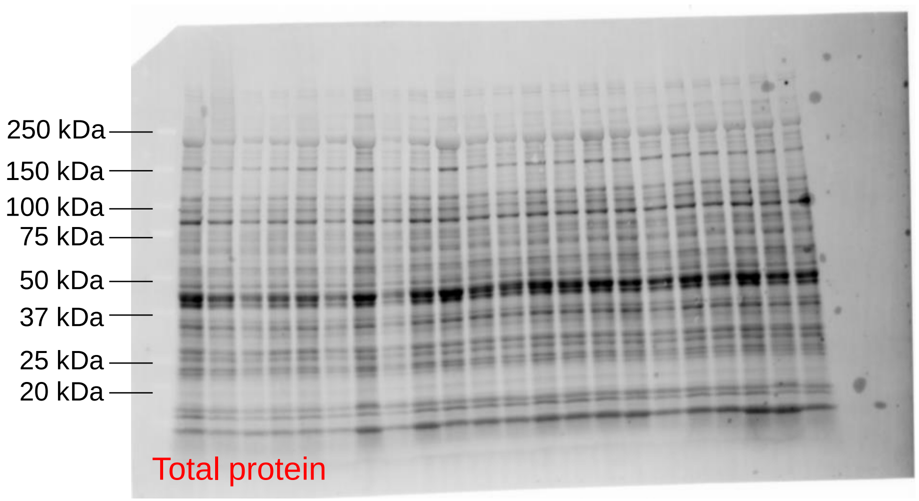

Disease Models & Mechanisms • Supplementary information

Fig. S4A

Set #2

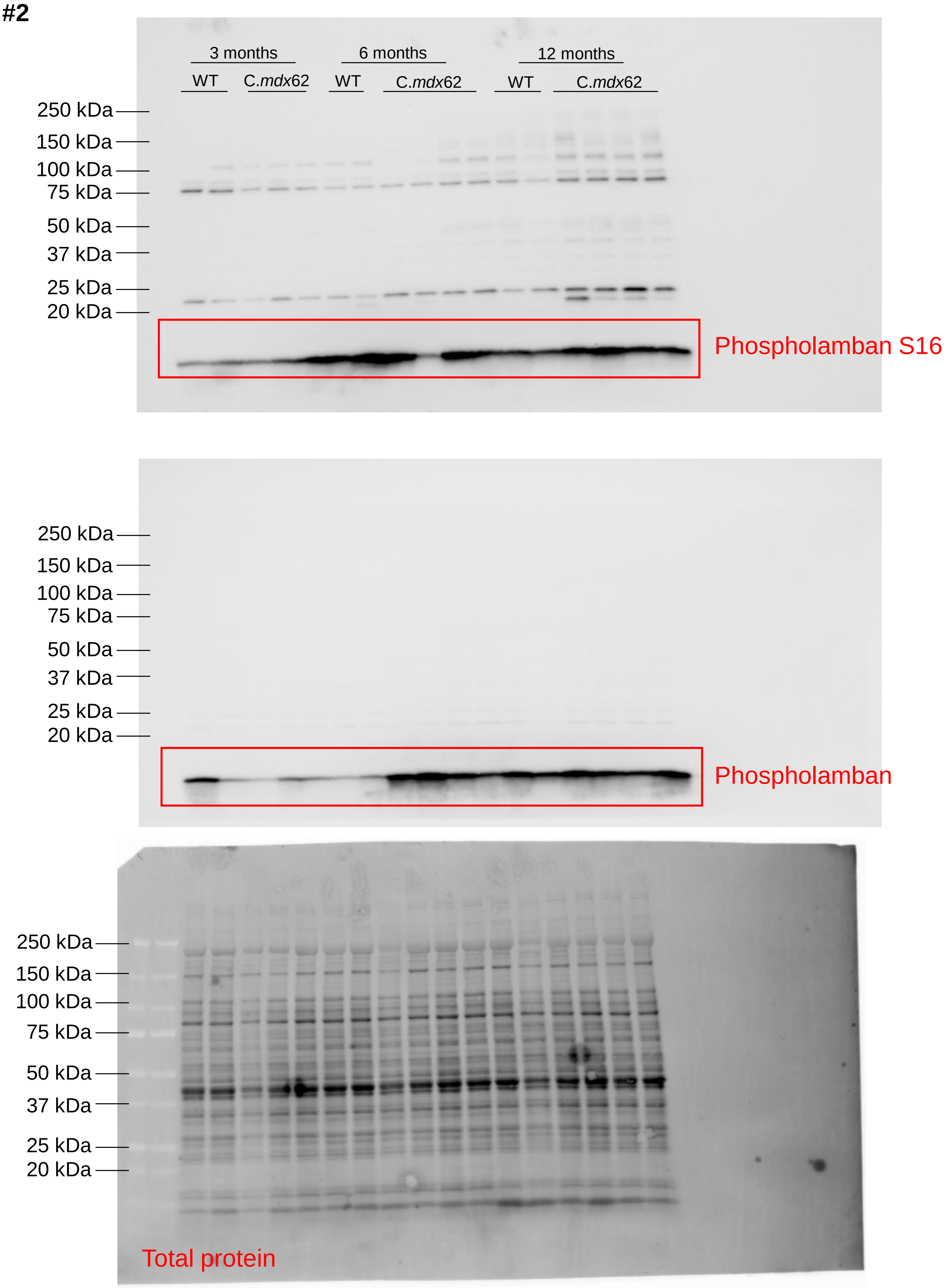

Disease Models & Mechanisms • Supplementary information
